# Seminars in epileptology: Presurgical epilepsy evaluation

**DOI:** 10.1002/epd2.70105

**Published:** 2025-09-24

**Authors:** Stephan Schuele, Robyn M. Busch, Birgit Frauscher, Vadym Gnatkovsky, Hajo Hamer, Lara Jehi, Andres Kanner, Georgia Ramantani, Theodor Rüber, Andreas Schulze‐Bonhage, Rainer Surges, Lara Wadi

**Affiliations:** ^1^ Department of Neurology Feinberg School of Medicine, Northwestern Memorial Hospital Chicago Illinois USA; ^2^ Department of Neurology Neurological Institute, Cleveland Clinic Cleveland Ohio USA; ^3^ Epilepsy Center Neurological Institute, Cleveland Clinic Cleveland Ohio USA; ^4^ Department of Neurology Duke University Medical Center and Department of Biomedical Engineering, Duke Pratt School of Engineering Durham North Carolina USA; ^5^ Department of Epileptology University Hospital Bonn Bonn Germany; ^6^ Department of Neurology, Epilepsy Center Friedrich‐Alexander‐University Erlangen‐Nuremberg Erlangen Germany; ^7^ Department of Neurology, Epilepsy Center University of Miami Miami USA; ^8^ Department of Neuropediatrics University Children's Hospital Zurich and University of Zurich Zurich Switzerland; ^9^ Epilepsy Center Neurocenter, University Medical Center, University of Freiburg Freiburg Germany; ^10^ Department of Neurology Duke University Medical Center Durham North Carolina USA

**Keywords:** etiology of surgical epilepsy, functional imaging, interictal epileptiform discharges, intracranial electroencephalography, non‐invasive electroencephalography, postoperative care, presurgical evaluation, presurgical neuropsychological assessment, rehabilitation, risk and benefit estimation, seizure onset pattern, stereoelectroencephalography, structural imaging

## Abstract

All patients with drug‐resistant seizures benefit from a comprehensive evaluation to confirm their seizure diagnosis and explore surgical treatment options. This seminar in epileptology discusses advancements in the field and provides specific didactic material to create an active working knowledge for the care of patients with focal drug‐resistant epilepsy. The article reviews indications for a presurgical evaluation and the importance and benefits of early surgical intervention. Advancements in diagnostic techniques in the presurgical evaluation, including video‐EEG monitoring, imaging, neuropsychological testing, and patient selection for invasive monitoring, are covered. An overview of common pathologies underlying surgical epilepsy syndromes and their MRI correlates is provided. A modern multimodal work‐up allows individualized risk and benefit estimation and a personalized approach to surgical decision‐making. The review concludes with a comprehensive discussion of postsurgical management, common complications, and rehabilitation after epilepsy surgery.

**Abbreviations**
ASM: anti‐seizure medication
BOLD: blood‐oxygen‐level‐dependent
DTI: Diffusion Tensor tensor Imagingimaging
EEG: electroencephalography
EMU: epilepsy monitoring unit
ESI: EEG source imaging
ETLE: extratemporal lobe epilepsy
FBTCS: focal to bilateral tonic‐–clonic seizure
FCD: focal cortical dysplasia
FDG: fluorodeoxyglucose
FMRI: functional magnetic resonance imaging
HD: high density
IED: interictal epileptiform discharge
IFCN: International Federation of Clinical Neurophysiology
ILAE: International leagueLeague againstAgainst epilepsyEpilepsy
ioECoG: intraoperative electrocorticography
ISAS: Ictal‐interictal SPECT Analysis using Statistical Parametric Mapping
LEAT: long‐term epilepsy‐associated tumor
LITT: laser interstitial thermal therapy
MEG: magnetoencephalography
MRI: magnetic resonance imaging
MSI: MEG source imaging
MSI/ESI: magnetic source imaging/Electrical source imaging
NAEC: National Association of Epilepsy Centers in the United States of America
PET: positron emission tomography
SCS: specific consistency score
SDE: subdural evaluation
SOZ: seizure onset zone
SPECT: single‐photon emission computed tomography
TL: temporal lobectomy
TLE: temporal lobe epilepsy

**Key points**

- A presurgical epilepsy evaluation is indicated for all patients with drug‐resistant epilepsy and should be offered without delay.
- Early epilepsy surgery, proven safe and effective, should be offered promptly, even in infancy, to enhance seizure control and developmental outcomes, and prevent the negative cognitive and social impacts of delayed intervention.
- Preoperative neuropsychological assessment is essential to evaluate cognitive and behavioral function, to make preoperative recommendations, and to aid with prediction of postsurgical cognitive and mood outcomes.
- Video‐EEG monitoring is the main pillar of a presurgical epilepsy evaluation besides imaging.
- Invasive implantation is indicated in patients where phase 1 presurgical evaluation does not allow proceeding directly to surgery, but where non‐invasive data still permit generating a strong hypothesis of a circumscribed epileptogenic zone network or focus which is potentially amenable to surgery.
- The presence of a structural lesion on MRI or localized hypometabolism on PET is independently associated with good postsurgical seizure outcome.
- Etiology determines not only the degree of drug resistance but also surgical outcomes and risk of resection.
- Individualized prediction of seizure, cognitive, and mood outcomes of epilepsy surgery is essential. Multiple tools now exist to assist in decision‐making, and technology offers the potential for new approaches.
- The epilepsy patient management conference is an integral part of the process providing optimal, consensus‐based care for the individual patient considered for surgical intervention.
- Risk factors for seizure recurrence after postoperative ASM taper include a short time between surgery and the start of withdrawal, IEDs on postoperative EEG, a longer duration of epilepsy, incomplete resection, the number of ASM at the time of surgery, and a history of FBTCS and seizures before withdrawal.
- De novo psychiatric disorders can develop in 10% to 15% of patients following a temporal lobectomy, the majority presenting as a mood and/or anxiety disorder and beginning between 3 and 6 months after surgery.

**Learning Objective of the International League Against Epilepsy Curriculum**

https://www.ilae.org/education/ilae‐curriculum.

4.1 Demonstrate working knowledge of indications for presurgical evaluation
4.2 Describe the importance of early surgical intervention regarding neurodevelopmental, cognitive, behavioral, and social integration aspects
4.3 Demonstrate working knowledge of advanced techniques for presurgical evaluation
4.4 Demonstrate working knowledge of etiologies amenable to surgical treatment and their prognoses in all age groups
4.5 Demonstrate ability to integrate information from multimodal work‐up and estimate risks and benefits from surgical therapy
4.6 Demonstrate working knowledge of postsurgical follow‐up
4.7 Demonstrate the value and need for multidisciplinary teamwork

This educational review paper addresses the learning objectives of the ILAE curriculum regarding epilepsy surgery.^1^ The concept of epilepsy surgery and the presurgical evaluation was discussed in a seminar's article in 2014.^2^ This article covers advancements in the field and provides specific didactic material to create active working knowledge for the surgical care of patients with drug‐resistant epilepsy (DRE). For an in‐depth discussion of invasive EEG interpretation, we refer to a recent publication in this seminar series.^3^

## 1 INDICATION FOR PRESURGICAL EVALUATION

The selection for a presurgical evaluation begins with the initial evaluation of any patient with suspected seizures.^4^ The initial history and witness report, search for prior events, frequency, family history, and risk factors help to confirm not only the diagnosis of epilepsy but also often provide localizing information and the suspicion for a focal epilepsy syndrome.^5^ Systematic use of epilepsy MRI imaging and interictal EEG in a first seizure clinic reveals an epileptogenic structural abnormality in around 25% of patients and a similar yield of focal epileptiform activity.^5^

Drug resistance is defined as “failure of adequate trials of two tolerated and appropriately chosen antiseizure medication (ASM) schedules (whether as monotherapies or in combination) to achieve seizure freedom”^6^ and has been considered a mandatory requirement for epilepsy surgery. Recent consensus recommendations confirmed that referrals for epilepsy surgery evaluation should be offered to every patient with drug‐resistant epilepsy (DRE).^7^ However, the panel also recommended that in patients with a brain lesion in non‐eloquent cortex, a surgical evaluation should be considered even if patients are seizure‐free on antiseizure medications (ASMs).

A surgical referral is cost effective, albeit less than 5% of patients end up being eligible for resective or ablative surgery.^8^ For many patients, the comprehensive evaluation, typically including an elective epilepsy monitoring unit (EMU) stay, provides diagnostic certainty, defines the epilepsy syndrome, explores the possibility of comorbidities, and helps the family and patient to understand seizure semiology and the accuracy of their self‐reported seizure count.^9^ For appropriately selected patients, epilepsy surgery not only improves seizure control, cognitive outcome, and quality of life, but also reduces mortality and improves survival in patients with DRE.^10^, ^11^

**FIGURE 1:**
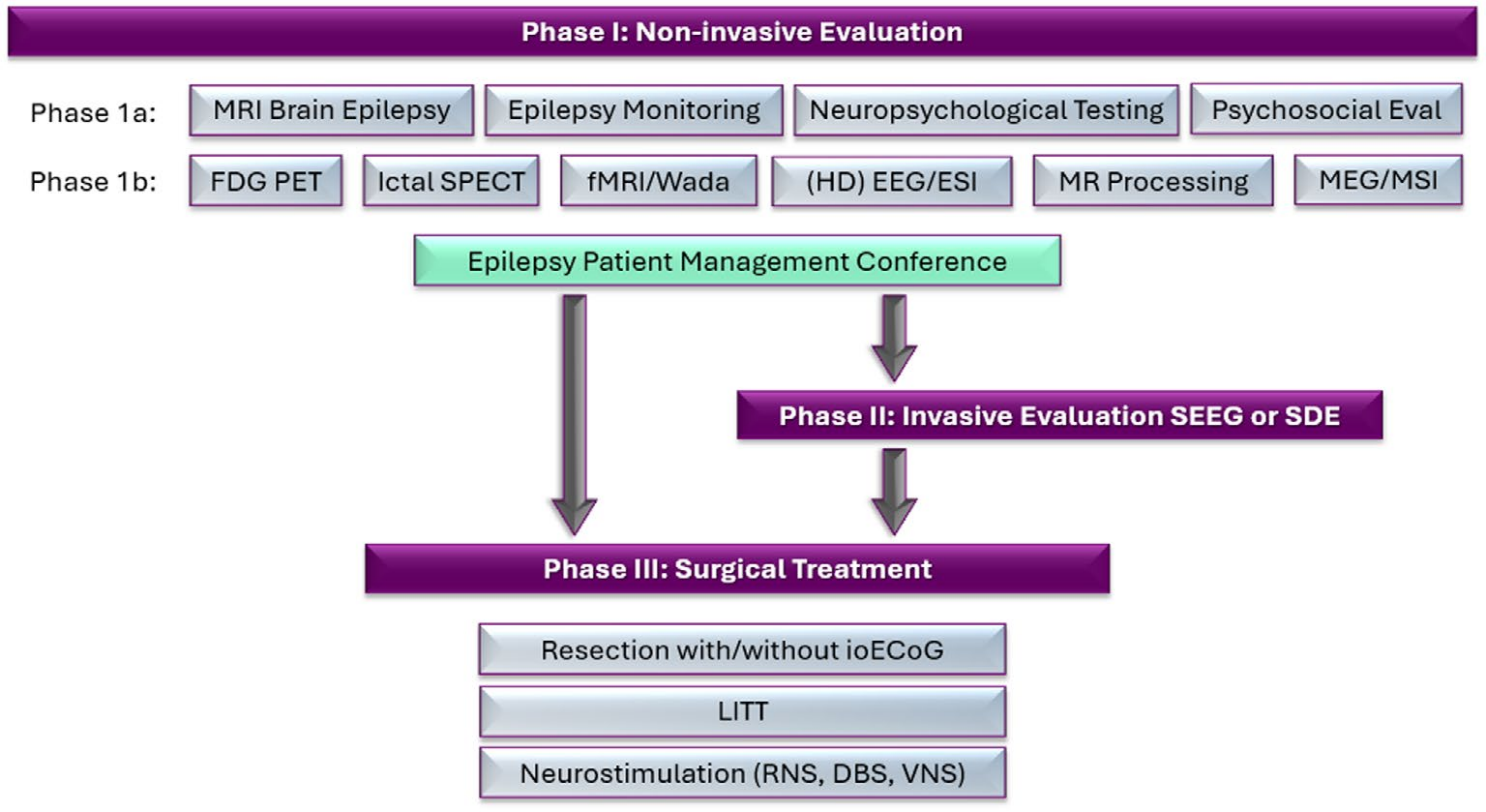
Epilepsy Patient Management Conference and Presurgical Epilepsy Evaluation Phase I–III. HD, high density; ESI, EEG source imaging; MSI, MEG source imaging; SDE, subdural evaluation.

The presurgical evaluation aims to delineate the epileptogenic zone or epileptogenic zone network, defined as the area of cortex to be removed or disconnected to render the patient seizure‐free.^12^, ^13^, ^14^, ^15^ This is accomplished by determining the symptomatogenic, irritative, ictal onset, and functional deficit zones and the epileptogenic lesion and eloquent cortex (see Table 1). In most surgical patients, this can be achieved through a non‐invasive Phase 1 evaluation (Figure 1), obtaining a detailed clinical history and examination, an epilepsy protocol MRI, neuropsychological testing, and an inpatient video‐EEG recording, and if necessary, complemented by PET, SPECT, fMRI, Wada testing, and electrical (ESI) or magnetic source imaging (MSI). In selected patients with concordant semiology, interictal EEG, and lesion site on MRI, direct referral for surgical resection without an inpatient video‐EEG recording of their seizures can be considered. Home video recording can help to confirm seizure semiology and prevent inadvertent surgery for non‐epileptic spells, which is one major concern when omitting inpatient video‐EEG recording.^16^ Genetic evaluation is increasingly advocated prior to epilepsy surgery and may guide decision‐making about surgical candidacy.^17^, ^18^ In two thirds of patients, a Phase 2 invasive evaluation can be “skipped,” and the patient is able to proceed to Phase 3, the actual surgical resection or ablation.

**TABLE 1:** Description of cortical zones for epilepsy surgery.

Description of cortical zones for epilepsy surgery	
Epileptogenic zone	Region of cortex that can generate the clinical seizures. Removal or disconnection is necessary to achieve seizure freedom.
Irritative zone	Region of cortex that generates interictal epileptiform discharges Interictal scalp or invasive EEG, electrical or magnetic source imaging
Seizure onset zone	Region where the seizures originate Long‐term scalp EEG, ictal SPECT, or invasive recordings of patient's habitual seizures
Epileptogenic lesion	Structural lesion that is causally related to the epilepsy MRI epilepsy protocol
Symptomatogenic zone	Region of cortex that generates the initial seizure symptoms. Assessed by history, video scalp EEG and invasively through electrical stimulation mapping (ESM) and ictal recording
Functional deficit zone	Region of cortex that in the interictal period is functionally abnormal, as indicated by: Neurological examination, neuropsychological testing, interictal PET, non‐epileptiform EEG/MEG findings
Eloquent cortex	Region of cortex that is indispensable for defined cortical functions Anatomy, handedness, neuropsychological assessment, functional MRI, MEG, Wada, invasively with SSEP, ESM

## 2 IMPORTANCE OF EARLY SURGICAL INTERVENTION

Considering that epilepsy surgery has proven safe and effective from an early age, it is imperative to offer access to this treatment as early as possible to those who may benefit from it. Early intervention in epilepsy surgery can be addressed in two ways: 1) timely intervention, shortening the latency from epilepsy onset to presurgical evaluation and surgical intervention, and 2) offering epilepsy surgery early, including in the first years of life.^19^, ^20^ The need for referral, evaluation, and intervention in children with DRE early in the disease course was emphasized by the ILAE Pediatric Epilepsy Surgery Task Force almost 20 years ago.^21^ In the meantime, a growing body of evidence supporting early or “expedited” epilepsy surgery^19^, ^22^ has led to shorter latencies to surgical intervention in focal epilepsy, although referral paths remain underutilized. Recently, the ILAE Surgical Therapies Commission has moved one step further, supporting early referrals for presurgical evaluation for patients with a well‐defined brain lesion in non‐eloquent, low‐risk areas of the brain who are seizure‐free on 1–2 ASMs.^7^ While epilepsy surgery before drug resistance is still debated, this approach has been linked to significantly higher rates of seizure freedom (98–100% vs. 77–88%) and, most importantly, ASM freedom in non‐drug‐resistant compared with drug‐resistant patients (54% vs. 38%).^23^, ^24^ Epilepsy duration remains an important modifiable predictor of surgical outcomes.

The importance of early surgical intervention is further supported by: (1) the low likelihood of ASM response in those with early onset, particularly in etiologies such as FCD, where the failure of just one ASM predisposes to a considerably increased incidence and earlier development of drug resistance (OR 346; 95% CI 19.6–6100),^25^ (2) the negative impact of epilepsy duration on postsurgical seizure outcomes, irrespective of age at surgery, localization, or etiology,^26^, ^27^, ^28^ and (3) the chance to attain not only an improved quality of life (QoL) when seizure freedom has been achieved,^29^ but also considerable developmental gains in relation to ASM withdrawal.^30^, ^31^ By contrast, delayed surgery may: (1) decrease the chances of surgical success due to ongoing epileptogenic processes over a longer epilepsy duration, (2) perpetuate cognitive decline related to the adverse effects of seizures, subclinical EEG discharges, and ASM, particularly on the developing brain,^30^, ^32^ (3) miss the opportunity for superior reorganization and deficit compensation related to plasticity in early life, such as language relocation, which is more effective before age 6, and 4) fail to mitigate the lifelong impact of epilepsy on cognition, behavior, socialization, education, and vocation (see Table 2).

**TABLE 2:** Factors supporting early surgery and consequences of delay.

Factors supporting early surgery	Consequences of delay
Higher incidence of epilepsy in infants and toddlers	Decreased chances of surgical success due to ongoing epileptogenic processes
Epilepsy surgery proven safe and effective from a young age	Perpetuated cognitive decline related to seizures, epileptiform EEG discharges, and ASM, especially in developing brains
Higher rates of sustained seizure and ASM freedom in non‐drug‐resistant patients	Missed opportunity for superior reorganization and deficit compensation related to plasticity in early life
Improved quality of life (QoL) and developmental gains with early seizure freedom	Lifelong impact on cognition, behavior, socialization, education, and vocation
Early surgery viewed as disease‐modifying with excellent outcomes in seizure and ASM freedom	Increased long‐term epilepsy‐related morbidity and mortality

Epilepsy incidence is higher in infants and toddlers compared with later in life, and drug resistance in the presence of cortical malformations or perinatal ischemic scars manifests at an early stage. Unremitting seizures, continuous epileptic discharges, and polytherapy gravely impact the developing brain, in addition to the underlying etiology, often leading to developmental arrest or regression.^20^ Epilepsy surgery in early life is no longer the treatment of last resort but should be perceived as “disease‐modifying”^19^ considering the excellent outcomes in terms of seizure and ASM freedom and optimal developmental outcomes.^20^, ^33^, ^34^ Surgical risks in younger children undergoing more extensive resections or disconnections may deter surgeons from pursuing early intervention. However, recent studies show that epilepsy surgery during the first few months of life is not associated with higher mortality and morbidity than surgery in older children, provided it is performed in tertiary centers with pediatric expertise. The risks of performing major surgery, such as hemispheric procedures, on the delicate infant brain should be weighed against the risks of epilepsy‐related morbidity and mortality over the course of the disease.^35^

## 3 PRESURGICAL EVALUATION

### 3.1 Non‐invasive video‐EEG monitoring

#### 3.1.1 Management of patients in the epilepsy monitoring unit, technical aspects, and behavioral testing during seizures

No single test is more critical than video‐electroencephalography for presurgical evaluation. Video‐EEG monitoring is performed in an EMU consisting of designated beds where video and EEG data are captured for continuous monitoring by trained personnel.^36^ The purpose of video‐EEG monitoring is to capture habitual electroclinical seizures as an essential part of the presurgical epilepsy evaluation. Given this specific purpose, all personnel are required to be specifically trained in seizure safety, behavioral testing during seizures, and management of seizures and seizure complications.

Following recommendations of the International Federation of Clinical Neurophysiology (IFCN), the basic array for presurgical evaluation has 25 electrodes. This array contains in addition to the standard 10–20 electrode system the inferior temporal chain that is essential to cover the anterior and basal temporal lobes.^37^ In addition, many centers have adopted cardiorespiratory monitoring to improve seizure detection, enhance patient safety, and provide information on autonomic clinical symptoms, which may be contributory to localization of seizure foci.^38^ For technical aspects of basic scalp EEG and protocols for cardiorespiratory monitoring, we refer to Beniczky and Schomer^39^ and Talavera et al.^38^

Admissions to the EMU are, on average, 3–4 days for presurgical evaluation.^40^ To increase the yield to capture habitual seizures, ASM are gradually tapered following center‐specific protocols that are individualized to the needs of an individual patient and account for seizure, medication, and patient‐related issues. Tapering by 30–50% at the time of admission may be appropriate. Tapering prior to admission is usually avoided unless the patient is on multiple drugs that have long half‐lives. Gradual tapering is done to avoid non‐habitual seizures and complications from bilateral tonic–clonic seizures. Despite discontinuation of ASM and seizure activation maneuvers such as sleep deprivation or patient‐specific triggering factors, it is possible that no seizure or not enough seizures are recorded during a single EMU admission, requiring a second admission. In order to achieve 90% confidence for the absence of multifocal seizure, three seizures are needed when the pretest probability for multifocal epilepsy is 20%, seven seizures for a pretest probability of 50%, and nine seizures for a pretest probability of 80%.^41^ Consequently, repeat monitoring should be arranged. For some cases, ambulatory video‐EEG might be a useful alternative for patients who do not need tapering of ASM and detailed behavioral testing during their seizures. Current research is focused on optimizing the yield of an individual EMU admission, leveraging the fact that seizures do not occur randomly but in predetermined cycles.^42^

Careful assessment of seizure semiology is key for identifying the epileptogenic zone. Many of these features can only be captured when the EMU staff tests patients appropriately. Standardized protocols have been developed to accomplish the goal of systematic seizure testing. They usually assess various motor, sensory, cognitive, and behavioral signs as well as specific functions important for an individual patient's seizures.^43^

#### 3.1.2 Interpretation of video‐EEG monitoring

Interpretation of video‐EEG monitoring comprises description of the background EEG, the interictal EEG including both epileptiform and non‐epileptiform anomalies, as well as a careful electroclinical correlation between the simultaneous video and the scalp EEG during seizures. To make results comparable across centers, it is important to follow standardized terminology (see glossary of EEG terminology by Kane et al.^44^ and glossary of seizure semiology by the International League against Epilepsy by Beniczky et al.^45^).

#### 3.1.3 Background and interictal activity

Initial review of the long‐term EEG recording begins with the identification of the awake and sleep background activity, the presence of physiological variants, and artifacts.^46^ Background slowing and generalized slowing suggest a diffuse encephalopathy. Asymmetry of background activity may indicate an area of cortical dysfunction or the sequela of a prior craniotomy. Persistent regional slowing is often associated with an underlying structural lesion, whether MRI‐positive or not.

The continuous EEG is carefully screened for interictal epileptic and non‐epileptic anomalies. The IFCN defines an interictal epileptiform discharges (IEDs) as a waveform that fulfills at least 4 out of 6 of the following: (1) di‐ or tri‐phasic wave with sharp or spiky morphology (i.e., pointed peak); (2) different wave durations than the ongoing background activity; (3) asymmetry of the waveform, with a sharply rising ascending phase and a more slowly decaying descending phase, or vice versa; (4) the transient is followed by an associated slow after‐wave; (5) the background activity surrounding IEDs is disrupted by the presence of the IEDs; and (6) distribution of the negative and positive potentials on the scalp suggests a source of the signal in the brain corresponding to a radial, oblique, or tangential orientation of the source.^44^ A subsequent study validating these criteria provided class III evidence that fulfilling 5 of these criteria has high specificity and sensitivity for identification of IEDs.^47^ Following these criteria avoids over‐interpretation and misdiagnosis of epilepsy and helps to clearly differentiate IEDs from normal variants that can be mistaken as IEDs.^48^ To improve localization accuracy, it is recommended not only to carefully interpret the raw EEG data in different montages but also to take advantage of voltage maps.^49^ High‐frequency activity in the high gamma (~50–100 Hz), ripple (~80–250 Hz), and fast ripple (~250–500 Hz) range is highly specific markers of the epileptic network and can be recorded on scalp EEG with the appropriate filtering despite their low amplitude.^50^, ^51^ In children, focal HFOs in the gamma and ripple range showed a lower sensitivity but higher specificity and accuracy for the EZ compared with spikes.^51^

As a rule of thumb, bilateral independent IEDs have a negative surgical prognosis. The exception is patients with exclusively unilateral temporal lobe epilepsy with homogeneous seizure semiology and unilateral recorded seizures in which bitemporal IEDs are common and should not preclude from epilepsy surgery. Also, children often have often more widespread IEDs and can nevertheless be excellent surgical candidates.^20^ Absence of spikes during scalp video‐EEG monitoring affects less than 10% of patients with a confirmed diagnosis of DRE considered for surgery and is seen in cases of either small generators or generators localized deep in the brain.^52^ In these situations, the use of high‐density EEG with at least 64 electrodes may be helpful, and ictal recording of the patient's habitual events is crucial to rule out alternative diagnoses and to define the seizure onset zone.

Importantly, over the past years, interictal algorithms based on Artificial Intelligence were developed that achieve expert‐level performance in fully automated interpretation of routine EEG,^53^ but this has not been tested for long‐term video‐EEG monitoring and seizures. Their implementation in commercial software solutions may ease the time‐consuming process of reviewing hours and days of recordings in the future.

#### 3.1.4 Seizures

Analysis of seizures requires a careful examination of the temporal sequence of semiology as assessed in the video as well as correlation to the accompanying EEG changes. A useful teaching resource is the glossary of seizure semiology published by the ILAE that serves to standardize vocabulary used to describe seizures but also provides ample video examples for the different seizure types.^45^ In brief, signs can be lateralizing (right/left; dominant/non‐dominant) or localizing at a lobar or sublobar level. Current efforts are undertaken to perform systematic reviews and meta‐analyses of the different ictal signs as well as different anatomical localizations. A special issue on this topic is currently under publication in Epileptic Disorders (https://www.paros‐epilepsy.com).

The Specific Consistency Score (SCS) is a tool that may be helpful to assist clinicians in assessing if a patient is a good candidate for direct surgery after phase 1 evaluation.^54^ A score of 9 points or higher (grade A) is associated with predicting direct surgical resection with good postoperative seizure outcomes. By contrast, a score of 4 points or lower (grade C) is seen in patients with inconsistent findings in their phase I evaluation. A score of 5–8 (grade B) suggests partially consistent findings and potential candidacy for surgical resection.

**FIGURE 2:**
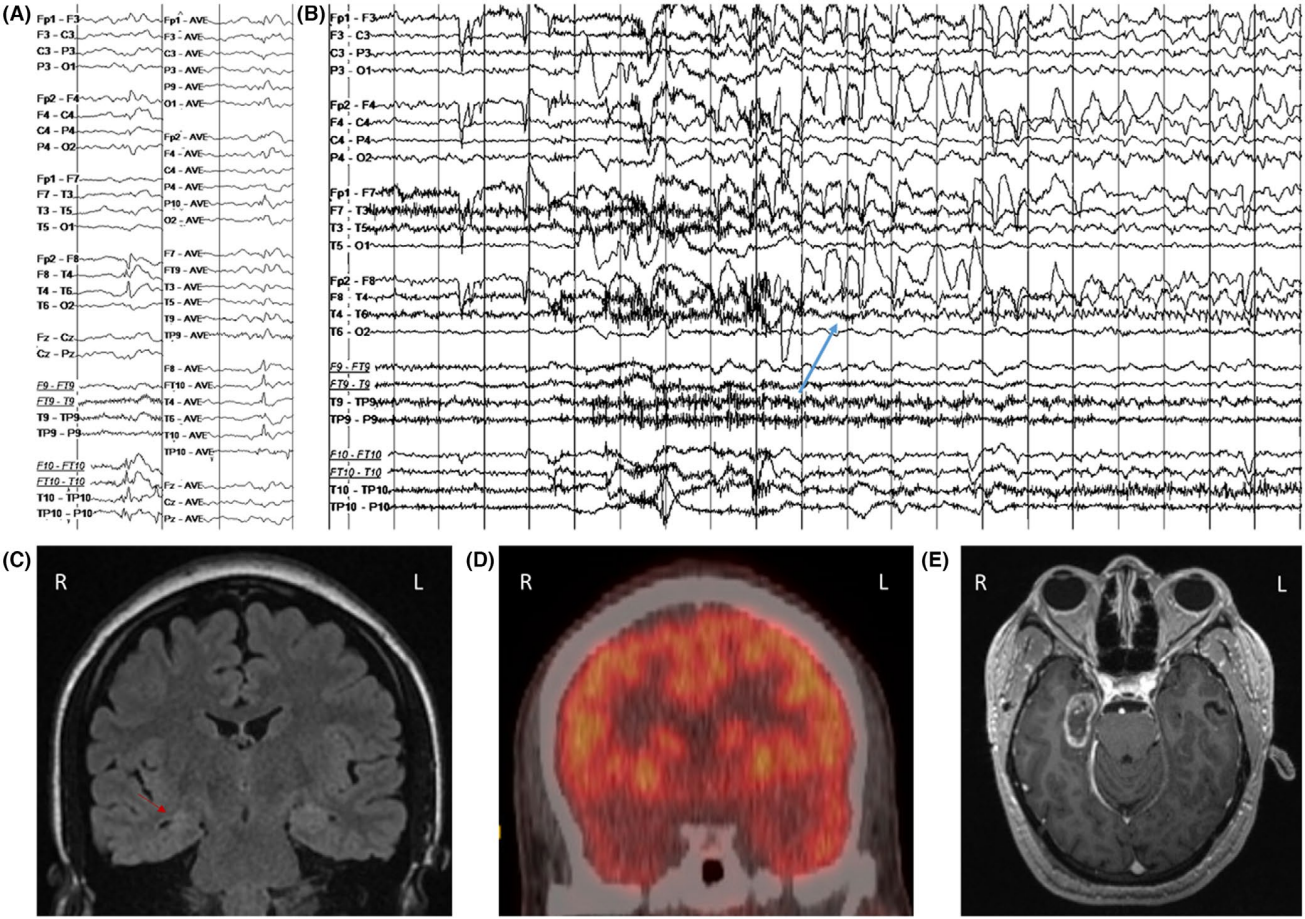
Example of a right mesiotemporal lobe epilepsy secondary to hippocampal sclerosis. Phase I work‐up (A–D) and surgical resection (E) in a 27‐year‐old ambidextrous woman with a family history of seizures in her paternal uncle and cousin. Epilepsy onset was at age 2 years with focal impaired consciousness seizures occurring multiple times per month and clustering around her menstrual period despite multiple antiseizure medications. Seizures were recorded as episodes of chest discomfort followed by vocal nonverbal automatisms (grunting) and distal flexor rotatory dystonic posturing of the left upper extremity with elementary distal automatisms of the right upper and right lower extremities, during which she did not respond to questions. In the immediate postictal phase, she was confused and fatigued but was able to communicate. (A) Interictal EEG showed frequent right anterior to mid‐temporal spikes; on average montage, the maximum was over electrodes F8, FT10, and T4. (1–70 Hz bandpass, bipolar longitudinal montage with subtemporal chains). Clinical onset (aura) preceded the electrographic onset, and (B) the first ictal changes on scalp EEG were characterized by rhythmic sinusoidal 5‐6 Hz theta activity over the right mid‐temporal region (arrow) (20s, 1–70 Hz bandpass, bipolar longitudinal montage with subtemporal chains). (C) 3 T brain MRI showed reduced right hippocampal volume with loss of internal architecture consistent with mesial temporal sclerosis (arrow), and (D) FDG‐PET showed right temporal hypometabolism. Neuropsychometric testing revealed deficits in learning and memory retrieval suggestive of temporal dysfunction but was not clearly lateralizing. Because the patient was ambidextrous, a functional language MRI (not shown here) was performed and revealed left hemisphere dominant expressive and receptive speech. Her overall phase I evaluation results were concordant with a right mesial temporal lobe epilepsy, and she was felt to be a good candidate for direct resection without additional invasive monitoring. As a result, the patient was offered a standard right anterior temporal lobectomy and right mesial temporal laser interstitial thermal therapy (LiTT). She opted for the latter and had an Engel IA outcome at her 1‐year follow‐up appointment. The laser ablation zone is shown in E. She continued to do well 4 years later with recurrence of rare non‐disabling auras (Engel IB). Her Specific Consistency Score was 11 (grade A), which supports that her data were highly consistent and that she was a suitable candidate for direct resection.

Figure 2 provides an illustrative case example of concordant findings in a phase 1 presurgical evaluation resulting in direct resective surgery.

**FIGURE 3:**
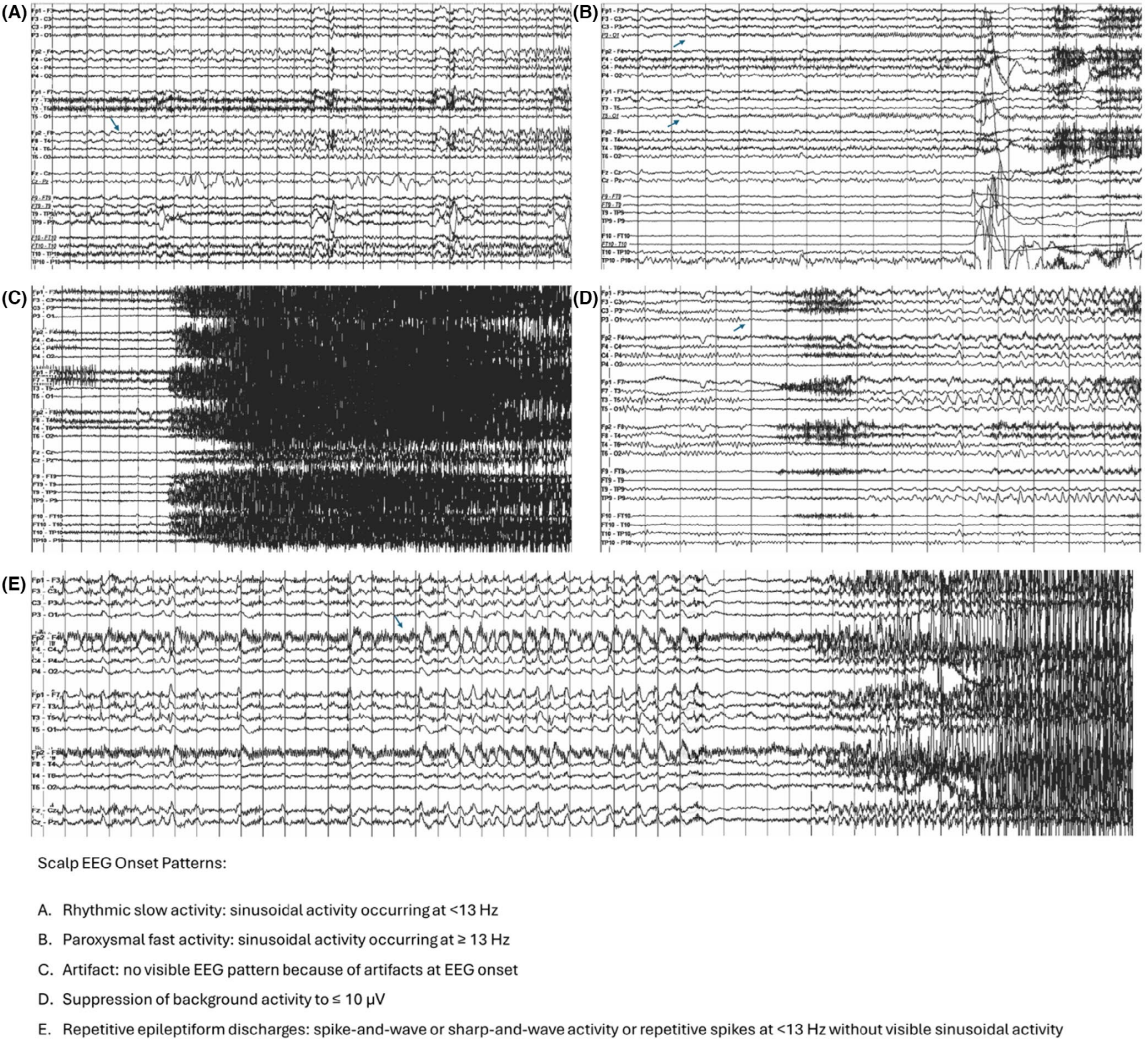
The most commonly described scalp EEG seizure onset patterns (Tanaka et al., 2018), with seizure onsets marked by an arrow (1–70 Hz bandpass, bipolar longitudinal montage). (A) Representative seizure onset in a 60‐year‐old right‐handed man with drug‐resistant epilepsy with focal impaired consciousness seizures starting with a viscerosensory aura (rising epigastric sensation) followed by behavioral arrest with oroalimentary automatisms (lip smacking and swallowing). Scalp EEG onset was characterized by 4–6 Hz rhythmic sinusoidal activity maximal over the right anterior to mid‐temporal region. His 3 T brain MRI showed right mesial temporal sclerosis, interictal FDG‐PET showed hypometabolism in the right anterior temporal lobe, and his neurocognitive profile was normal. The patient was offered a standard right anterior temporal lobectomy. (B) Representative seizure onset in a 25‐year‐old right‐handed woman with focal impaired consciousness seizures characterized by an elementary visual aura (seeing bright lights or colorful shapes described as “film of gasoline”) followed by confusion, often evolving into bilateral tonic–clonic seizures with a versive head turn to the right and right gaze deviation. Scalp EEG ictal onset was characterized by focal paroxysmal fast activity in the beta range maximal over the left occipital region (O1). 3T brain MRI was non‐lesional, FDG‐PET showed areas of hypometabolism in the left occipital and parietal regions, and neuropsychometric testing showed deficits in complex visual organization and spatial perception localizing to the left posterior quadrant. SEEG seizure onset (not shown here) was over the left infracalcarine occipital cortex, and a focal resection was offered to the patient. (C) Case of a 21‐year‐old right‐handed man with focal impaired consciousness seizures often evolving into bilateral tonic–clonic seizures, the majority of which were sleep‐related, characterized by facial grimacing, asymmetric tonic posturing of the left > right upper extremities, brief clonic jerks of the left upper extremity, followed by bilateral tonic–clonic activity. Scalp ictal onset was often non‐lateralizing and non‐localizing characterized by diffuse myogenic artifact coinciding with clinical onset. With some seizures, ictal onset was characterized by a focal attenuation of the background over the right anterior to mid‐temporal region (similar to EEG onset pattern #4—not shown here). 3T brain MRI was non‐lesional, FDG‐PET showed areas of hypometabolism in the right anterior temporal and right inferior frontal lobes, and his neurocognitive profile was normal. SEEG seizure onset was seen over the right anterior temporal neocortex and temporal pole, and resective surgery was offered to the patient. (D) Representative seizure onset in a 23‐year‐old right‐handed woman with focal preserved consciousness seizures characterized by an elementary visual aura (visual obscuration followed by seeing colorful shapes in the right visual hemifield) and frequent sleep‐related focal to bilateral tonic–clonic seizures with dystonic posturing of the right upper extremity followed by bilateral tonic–clonic activity. Scalp EEG ictal onset was characterized by focal suppression of the background to <10 μV seen over the left occipital region. 3T brain MRI showed hyperintense T2/FLAIR signal in the left mesial occipital lobe suggestive of a possible focal cortical dysplasia, FDG‐PET revealed an area of hypometabolism within the left occipital and posterior temporal region, and neuropsychometric testing showed deficits in complex visuospatial organization and in verbal skills, suggesting dominant posterior quadrant dysfunction. SEEG confirmed seizure onset (not shown here) within the left infracalcarine occipital cortex, and the patient opted for a responsive neurostimulation (RNS) device as opposed to resective surgery to minimize the risk of a visual field deficit. (E) Representative seizure onset in a 34‐year‐old right‐handed woman with sleep‐related focal to bilateral tonic–clonic seizures characterized by staring followed by versive head turn to the left and left gaze deviation at onset then by a fencer posture (right arm flexion, left arm extension), with no identifiable aura. Interictal scalp EEG showed near continuous bilateral synchronous spike wave discharges over the anterior head region, and ictal onset was characterized by rhythmic repetitive 1.5 Hz spike wave discharges with maximum negativity over the right frontal/frontopolar region (arrow) extending to the right central region with rapid secondary bilateral synchrony. 3 T brain MRI was non‐lesional, FDG‐PET showed hypometabolism in the right frontotemporal region, and neuropsychometric testing suggested non‐lateralized frontal lobe dysfunction. SEEG seizure onset (not shown here) was seen over the right orbitofrontal region with rapid propagation to the right temporal pole, and resective surgery was offered to the patient.

Figure 3 illustrates the 5 most common scalp seizure onset patterns as described in the literature.^55^

Scalp seizure onset patterns were found useful to separate neocortical from mesial temporal generators. The typical ictal scalp pattern for a seizure involving the hippocampus is a 5–7 Hz rhythmic theta activity over the temporal leads. Neocortical seizures usually begin as focal or lateralized delta activity, beta activity, low voltage fast, or lateralized suppression.^56^ Frontal seizures tend to be brief and have a tendency for rapid spread; hence, they are more difficult to localize than temporal seizures. One main reason for failed anterior temporal lobe epilepsy surgery is temporal plus epilepsy,^57^ defined as a primary temporal lobe epileptogenic zone extending to neighboring regions. A red flag for temporal plus epilepsies is that first ictal EEG changes were more frequently localized over the anterior frontal, temporo‐parietal, or the precentral region compared to patients with anterior temporal lobe epilepsy.

**FIGURE 4:**
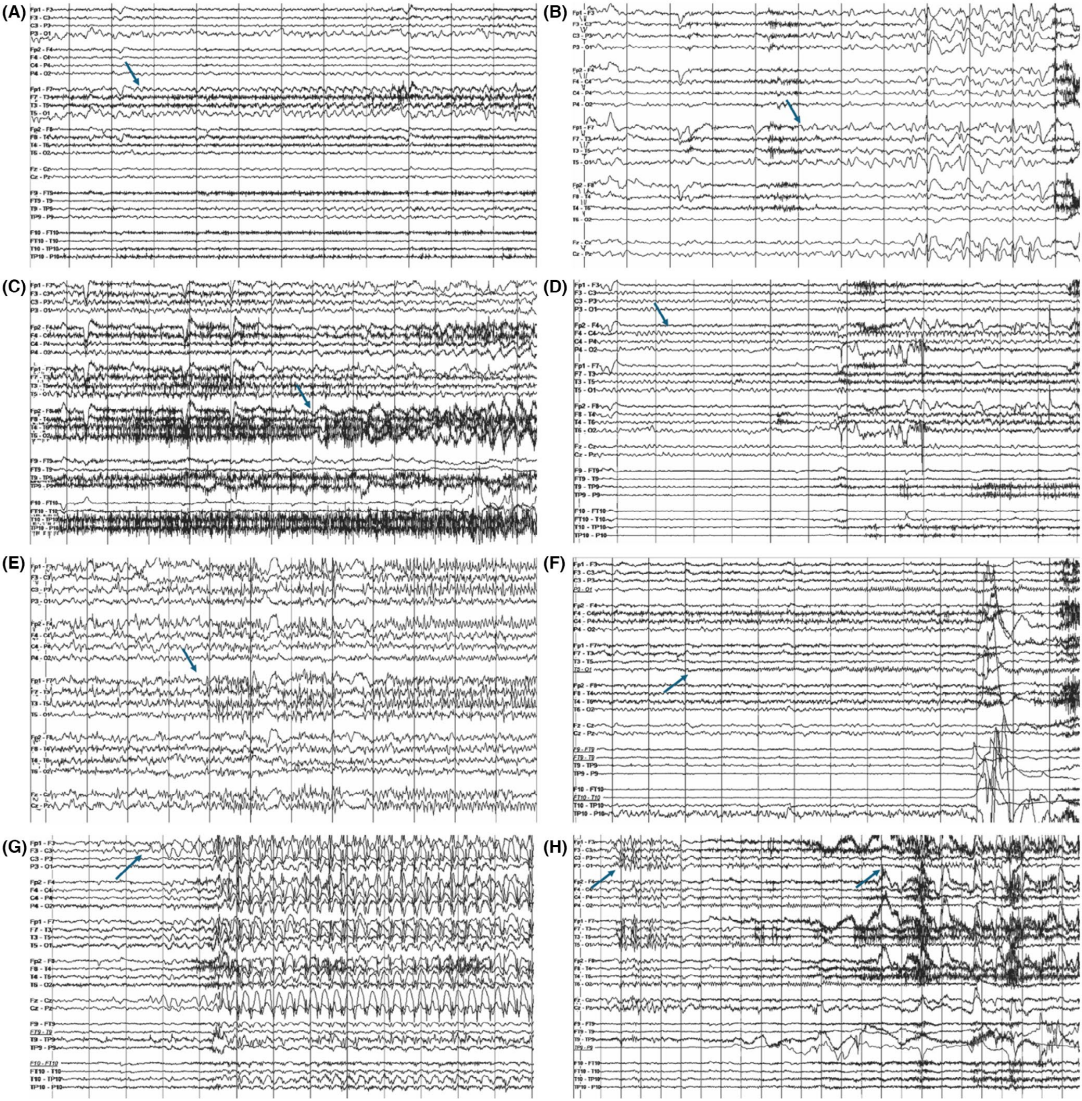
Seizure onset pattern by anatomical region, with seizure onsets marked by an arrow (1–70 Hz bandpass, bipolar longitudinal montage). (A) Representative seizure onset pattern in a case of mesial temporal lobe epilepsy. 34‐year‐old right‐handed woman with focal impaired consciousness seizures characterized by a viscerosensory aura (rising epigastric sensation), ictal tachycardia, followed by oroalimentary automatisms (chewing and swallowing movements) and staring, postictal aphasia, with occasional focal to bilateral tonic–clonic seizures. Scalp EEG ictal onset was characterized by sinusoidal rhythmic 5‐6 Hz activity maximal over the left anterior to mid‐temporal region. 3T brain MRI showed left mesial temporal sclerosis, FDG‐PET showed an area of hypometabolism over the left anterior temporal region, and neuropsychological testing revealed deficits in verbal learning and memory consistent with dominant temporal dysfunction. The patient was offered a left anterior temporal lobectomy. (B) Representative seizure onset pattern in a case of neocortical temporal lobe epilepsy. 38‐year‐old right‐handed man with focal preserved consciousness seizures characterized by mixed receptive>expressive aphasia (he has difficulty understanding what people are saying to him and can only communicate in simple sentences) and difficulty reading, occasionally evolving to bilateral tonic–clonic seizures with right gaze deviation and versive head turn to the right. Scalp EEG ictal onset was characterized by irregular 2.5‐4 Hz polymorphic slow discharges over the left temporal region. 3T brain MRI was non‐lesional, and his FDG‐PET and neurocognitive profile were non‐lateralizing or localizing. SEEG (not shown here) seizure onset was seen over the left posterior superior temporal gyrus with rapid propagation to the left basal temporal language area. Seizure onset co‐localized with eloquent language cortex, and neuromodulation with a responsive neurostimulation (RNS) device was offered. (C) Representative seizure onset pattern in a case of neocortical temporal lobe epilepsy. 31‐year‐old right‐handed man with focal impaired consciousness seizures characterized by staring and behavioral arrest in the absence of an aura, often with rapid evolution to bilateral tonic–clonic activity with leftward versive head turn and gaze deviation. Scalp EEG onset was characterized by slow discharges in the delta‐range maximal over the right temporal region. 3T brain MRI was non‐lesional, FDG‐PET was non‐lateralizing or localizing, and neuropsychological evaluation showed deficits in working memory, processing speed, and visual‐motor integration. SEEG ictal onset (not shown here) was seen over the right middle and posterior superior temporal gyrus, and the patient was offered a surgical resection. (D) Representative seizure onset pattern in a case of temporal lobe plus epilepsy. An 11‐year‐old right‐handed girl with focal motor seizures with preserved consciousness characterized by contractions of the tongue resulting in speech impairment followed by left‐sided perioral clonic activity, rarely with evolution to bilateral tonic–clonic activity. Scalp EEG onset was characterized by focal attenuation of the background followed by sharply contoured rhythmic theta activity over the right frontal region (maximal over F4) with rapid propagation to the right central and temporal regions. 3T brain MRI showed a large cavernoma in the right anterior temporal lobe extending into the right frontal opercular region. Neuropsychological evaluation revealed deficit in planning and organization pointing to non‐dominant frontal lobe dysfunction. Functional MRI for language evaluation showed left hemisphere dominant expressive and receptive speech. Surgical resection of the cavernoma was offered to the patient. (E) Representative seizure onset pattern in a case of frontal lobe focal cortical dysplasia. 20‐year‐old right‐handed woman with focal motor seizures with impaired consciousness characterized by versive head turn to the right with right sided perioral and periorbital clonic activity, occasionally with clonic activity of the right upper extremity. Scalp EEG onset was characterized by rapid repetitive spiking maximal over the left frontal region with rapid hemispheric involvement. 3T brain MRI showed an area of cortical thickening and irregularity with blurring of the gray–white matter junction in the left inferior frontal lobe suggestive of a focal cortical dysplasia. Neuropsychometric testing showed deficits in attention and executive functioning suggestive of non‐lateralizing frontal lobe dysfunction, and FDG‐PET showed areas of hypometabolism in the left anterior temporal and frontal regions. (F) Representative seizure onset pattern in a case of occipital lobe epilepsy. 25‐year‐old right‐handed woman with focal impaired consciousness seizures characterized by an elementary visual aura (seeing bright lights or colorful shapes described as “film of gasoline”) followed by confusion, often evolving into bilateral tonic–clonic seizures with a versive head turn to the right and right gaze deviation. Scalp EEG ictal onset was characterized by focal paroxysmal fast activity in beta range maximal over left occipital region (O1). 3T brain MRI was non‐lesional, FDG‐PET showed areas of hypometabolism in the left occipital and parietal regions, and neuropsychometric testing showed deficits in complex visual organization and spatial perception localizing to the left posterior quadrant. SEEG seizure onset (not shown here) was over the left infracalcarine occipital cortex, and a focal resection was offered to the patient. (G) Representative seizure onset pattern in a case of frontal lobe epilepsy with rapid bilateral synchrony. 13‐year‐old right‐handed male with focal motor seizures with impaired consciousness and focal to bilateral tonic–clonic seizures characterized by asymmetric tonic posturing of the upper extremities at onset, occasionally with right sided perioral and periorbital facial clonic activity. Scalp EEG onset was characterized by rhythmic spike and slow wave discharges with bifrontal predominance and rapid bilateral synchrony, occasionally with a lead‐in over the left frontal region and midline (arrow). 3T brain MRI showed blurring of the gray–white matter junction in the left frontal operculum suggestive of a focal cortical dysplasia. Neuropsychometric testing showed inefficiency in working memory, verbal fluency, and rote verbal learning suggesting dominant frontal lobe dysfunction. FDG‐PET showed areas of hypometabolism in the left frontal operculum and temporal lobe. (H) Representative seizure onset pattern in a case of posterior cortex epilepsy. 27‐year‐old right‐handed woman with focal impaired consciousness seizures characterized by painful sensation in the left upper extremity followed by genital automatisms with the right hand and confusion, and focal preserved consciousness seizures characterized by diaphoresis, tachycardia, and tingling of the left palm. Scalp EEG onset was characterized by heralding spikes over the left posterior temporal–parietal region with subsequent sharply contoured rhythmic theta activity over the left posterior quadrant. 3T brain MRI was non‐lesional, FDG‐PET showed an area of hypometabolism in the left anterior temporal lobe, and neurocognitive profile was normal. Intracranial monitoring suggested a posterior cortex epilepsy with seizure onsets seen over the left parietal region and left posterior superior temporal gyrus suggestive of a network organization widespread over the posterior cortex, and resulting in a recommendation for neuromodulation with an RNS device.

Examples for ictal patterns for frequent epilepsy types are provided in Figure 4.

### 3.2 Structural and functional neuroimaging

Various multimodal imaging techniques are in clinical use to generate hypotheses on the localization of the epileptogenic zone and to identify eloquent brain areas, including brain MRI, single‐photon emission computed tomography (SPECT), and positron emission tomography (PET).

#### 3.2.1 Cerebral MRI

The gold standard for the identification of epileptogenic lesions is the brain MRI. The MRI should be reviewed in the context of an electroclinical hypothesis to improve the yield of correctly identifying the often subtle abnormalities associated with DRE. An imaging protocol for people with epilepsy should provide both optimal volumes for visual assessment and ideal input data for postprocessing (Table 3).^58^, ^59^

**TABLE 3:** MRI routine protocol optimized for individuals with epilepsy.

Sequence	Cut‐plane orientation	Cut‐plane angulation
3D T1	Three‐dimensional	ac‐pc
3D FLAIR	Three‐dimensional	ac‐pc
T2	Coronal	ac‐pc^a^/lha^b^
FLAIR	Coronal	ac‐pc^a^/lha^b^
T2	Axial	ac‐pc^a^/lha^b^
FLAIR	Axial	ac‐pc^a^/lha^b^
Hemo/Calc	Axial	ac‐pc

*Note*: MRI routine protocol optimized for individuals with epilepsy.

Abbreviations: ac‐pc, anterior commissure‐posterior commissure line; Hemo/Calc, hemosiderin/calcification‐sensitive sequence; lha, long hippocampal axis.

^a^ In individuals with suspected extratemporal epilepsy, angulation of volumes should be oriented to the anterior commissure‐posterior commissure line.

^b^ In individuals with suspected temporal epilepsy, angulation of volumes should be oriented to the hippocampal long axis.

Different protocols for individuals with suspected temporal lobe epilepsy (TLE) and suspected extratemporal lobe epilepsy (ETLE) are recommended. Both groups should undergo first T1 and FLAIR in isotropic resolution (“three‐dimensional volumes” with isotropic voxels that have the same nominal resolution along all three spatial axes). The resulting volumes may then be used to plan angulation for the subsequent two‐dimensional sequences T2 and FLAIR (with anisotropic voxels that show low resolution along only two axes, usually coronal and axial).^60^ In suspected TLE, axial slices should be angulated collinear to the hippocampal long axis and coronal slices perpendicular to it. The structure of the hippocampal formation's internal morphology can only be fully evaluated using two‐dimensional sequences that are precisely aligned with the long axis of the hippocampus. In suspected ETLE, two‐dimensional sequences should be angulated along the anterior–posterior commissure line. Lastly, a hemosiderin/calcification‐sensitive sequence is obligatory in every epilepsy protocol to detect small arteriovenous malformations or cavernomas. Contrast‐enhanced sequences may be needed to further investigate the nature of a lesion (e.g., type of tumor or vascular lesion) but are often not routinely acquired.^58^, ^61^ In general, FLAIR is the sequence with the highest sensitivity.^58^

**FIGURE 5:**
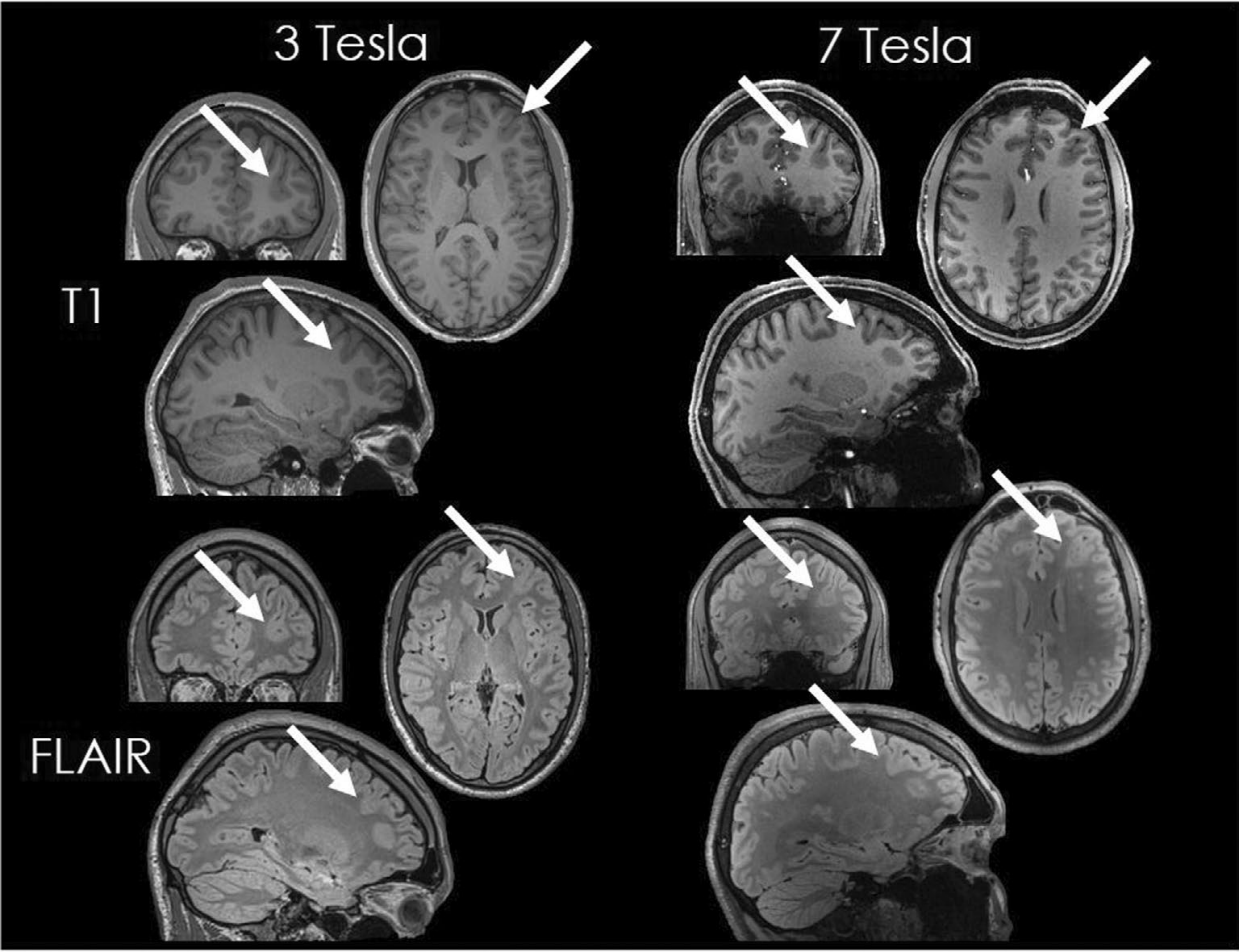
3D‐T1 and 3D‐FLAIR weighted sequences using 3 Tesla (left images) and 7 Tesla (right images) of a 40‐year‐old male patient with focal cortical dysplasia in the left frontal region.

An important interpretative pitfall is the presence of transient, peri‐ictal signal abnormalities. Following prolonged or frequent seizures (especially status epilepticus), focal hyperintensity on FLAIR and diffusion alterations can appear in the cortex, subcortical white matter, or hippocampus.^62^, ^63^, ^64^ These changes reflect peri‐ictal vasogenic and cytotoxic edema and can mimic structural lesions like focal cortical dysplasia or encephalitis. While 3 Tesla was considered the gold‐standard field strength in epilepsy imaging, the advent of 7 Tesla MRI has raised the hope of decreasing the number of MR‐negative individuals (Figure 5).^65^, ^66^ However, apart from the limited availability, it should still be recognized that a 7 T scanner is not only an enhanced version of a 3 T scanner, but it also comes along with distinct challenges including field inhomogeneities and incompatibilities with subjects.^67^, ^68^

Generally, the diagnostic yield of neuroimaging in epilepsy is critically dependent on a multidisciplinary approach. MRI studies should not be considered in isolation. Crucially, the interpreting radiologist must be informed of the electroclinical hypothesis, including seizure semiology and EEG findings, which suggest a potential seizure onset zone. This clinical context guides a targeted “second look” at the images, which can reveal subtle abnormalities that might otherwise be overlooked.^69^ It is imperative that they are performed using a dedicated epilepsy protocols and interpreted by a neuroradiologist with expertise in epilepsy.^58^ This is even more important in infants and toddlers,^70^ primarily due to the dynamic process of brain myelination. The characteristic lack of contrast between gray and white matter in neonates and young infants can obscure or mimic abnormalities of cortical development. The gray–white matter junction, a key area for detecting focal cortical dysplasias, is often indistinct.^70^ Furthermore, normal developmental stages of myelination can be mistaken for pathology. Therefore, MRI studies in this age group must be interpreted by a neuroradiologist with specific pediatric expertise, using age‐appropriate imaging protocols and atlases to avoid misdiagnosis.

**FIGURE 6:**
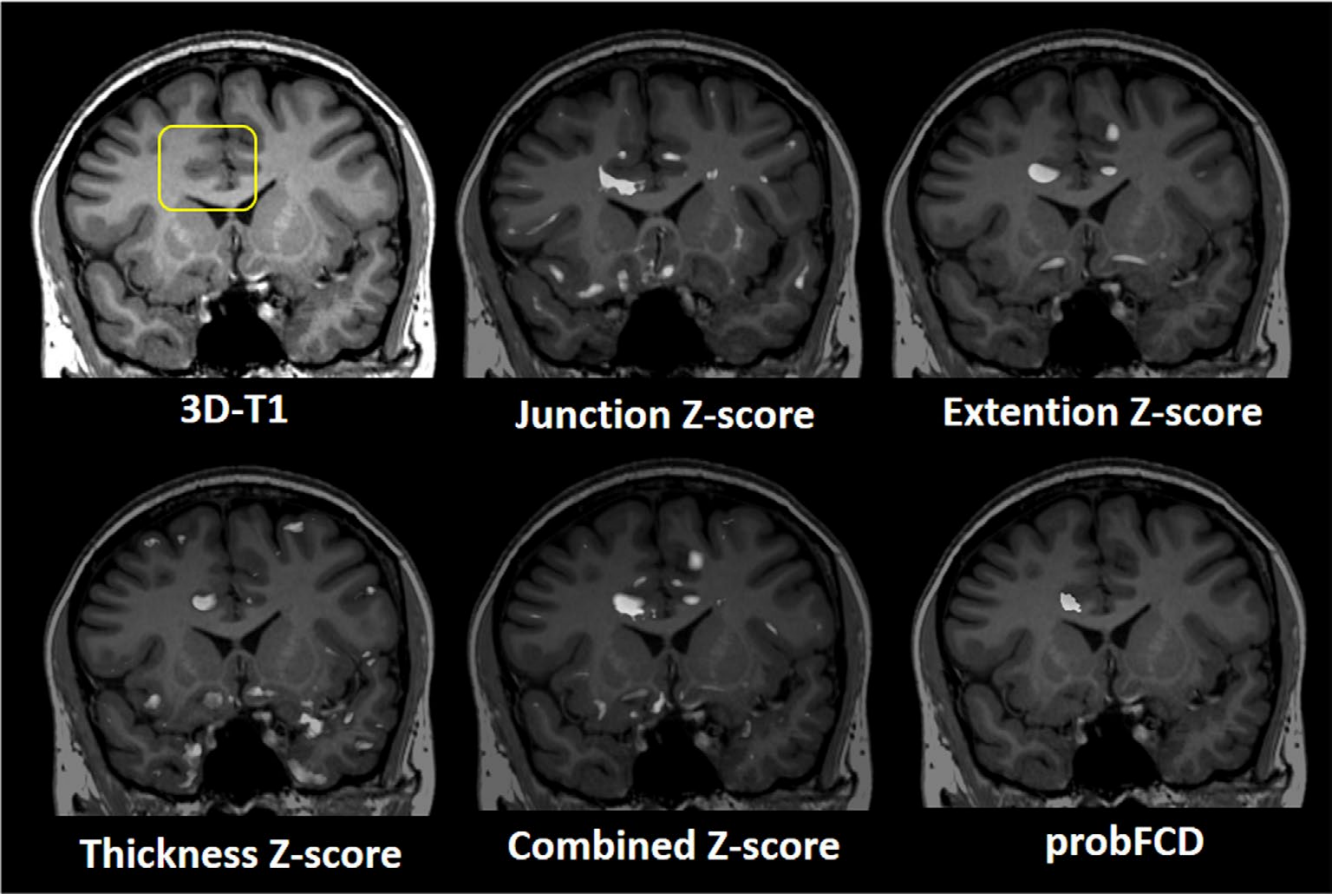
3D‐T1 and principal MAP analysis scores of a 36‐year‐old female patient. Initially reported as MRI‐negative, the patient was reclassified as MRI‐positive following postprocessing of structural images. Focal cortical dysplasia (FCD IIa) was confirmed histopathologically after SEEG‐guided resection. The FCD suspected area is marked by a yellow rectangle.

Three‐dimensional, isotropic volumes are not only needed for anatomical orientation, as they enable reconstruction of images in any plane, but also as input data for postprocessing, which is used as a complement to standard visual assessment in tertiary epilepsy centers. MRI postprocessing operates hypothesis‐free, operator‐independent, and three‐dimensionally, which is why it can simultaneously consider structural features from different slices and detect pathological entities that elude visual assessment. However, no postprocessing routines can be universally applied without question because of the absence of systematic evaluation and the widespread reliance on in‐house software.^71^ Parcellations and quantitative signal assessments of mesial temporal structures as well as voxel‐wise morphometric MRI analyses for lesion detection are the most used postprocessing routines in epilepsy imaging.^72^, ^73^, ^74^ The Morphometric Analysis Program (MAP) is the only program that has earned a place in the clinical routine of dozens of centers worldwide for its utility in detecting subtle focal cortical dysplasias.^75^ MAP is a voxel‐based method that compares the patient's T1‐weighted MRI to a large normative database of healthy controls. It generates statistical z‐score maps that highlight areas of abnormal cortical thickness or blurring of the gray–white matter junction features characteristic of focal cortical dysplasias (see Figure 6). These quantitative maps can guide the radiologist to subtle lesions that were occult on visual inspection of conventional MRI slices, thereby increasing the diagnostic yield in patients previously classified as “MRI‐negative”.^73^ Emerging deep learning techniques for detecting subtle epileptogenic lesions such as MELD^76^ show significant promise and are poised to serve as a powerful complement to established methods like MAP. Beyond structural imaging, perfusion sequences provide valuable functional information. Arterial Spin Labeling (ASL) is a non‐invasive perfusion MRI technique that does not require an exogenous contrast agent.^77^ It can help localize the seizure onset zone by detecting characteristic blood flow changes: typically, postictal hypoperfusion (decreased blood flow). The ability to co‐register ASL data with anatomical T1‐weighted images makes it a powerful tool for localizing epileptogenic zones, particularly in MRI‐negative cases, serving as a non‐radioactive alternative to SPECT or even PET scans.^78^

#### 3.2.2 Single‐photon emission computed tomography (SPECT)

Ictal SPECT is the only clinically established imaging modality for the visualization of single epileptic seizures, while the rest of epilepsy imaging is geared towards identifying a potentially epileptogenic structural lesion or area of dysfunction.^60^ Increased cerebral blood flow near the seizure onset zone during epileptic seizures can be visualized using ^99m^Technetium‐labeled radiopharmaceuticals injected shortly after the seizure onset.^79^ The injection of radiopharmaceuticals is repeated in a seizure‐free interval, and the SPECT images acquired after ictal and interictal injection are compared. While ictal SPECT is resource‐intensive and involves radiation exposure, it can provide valuable insights. The efficacy of ictal SPECT varies with the seizure onset location, showing higher localization success in TLE compared with ETLE.^80^, ^81^ Advanced postprocessing techniques, such as ictal‐interictal SPECT Analysis using Statistical Parametric Mapping (ISAS), statistically evaluate the differences between the ictal image and an additional interictal image, comparing them with scan‐to‐scan variations in healthy controls to delineate regions of significant ictal hyperperfusion.^82^ The evidence of an added value of ictal SPECT in clinical decision‐making is rather mixed.^80^, ^83^ In well‐selected individuals with inconclusive findings, however, ictal SPECT can provide additional information to guide further intracranial EEG or surgery.

#### 3.2.3 Positron emission tomography (PET)

**FIGURE 7:**
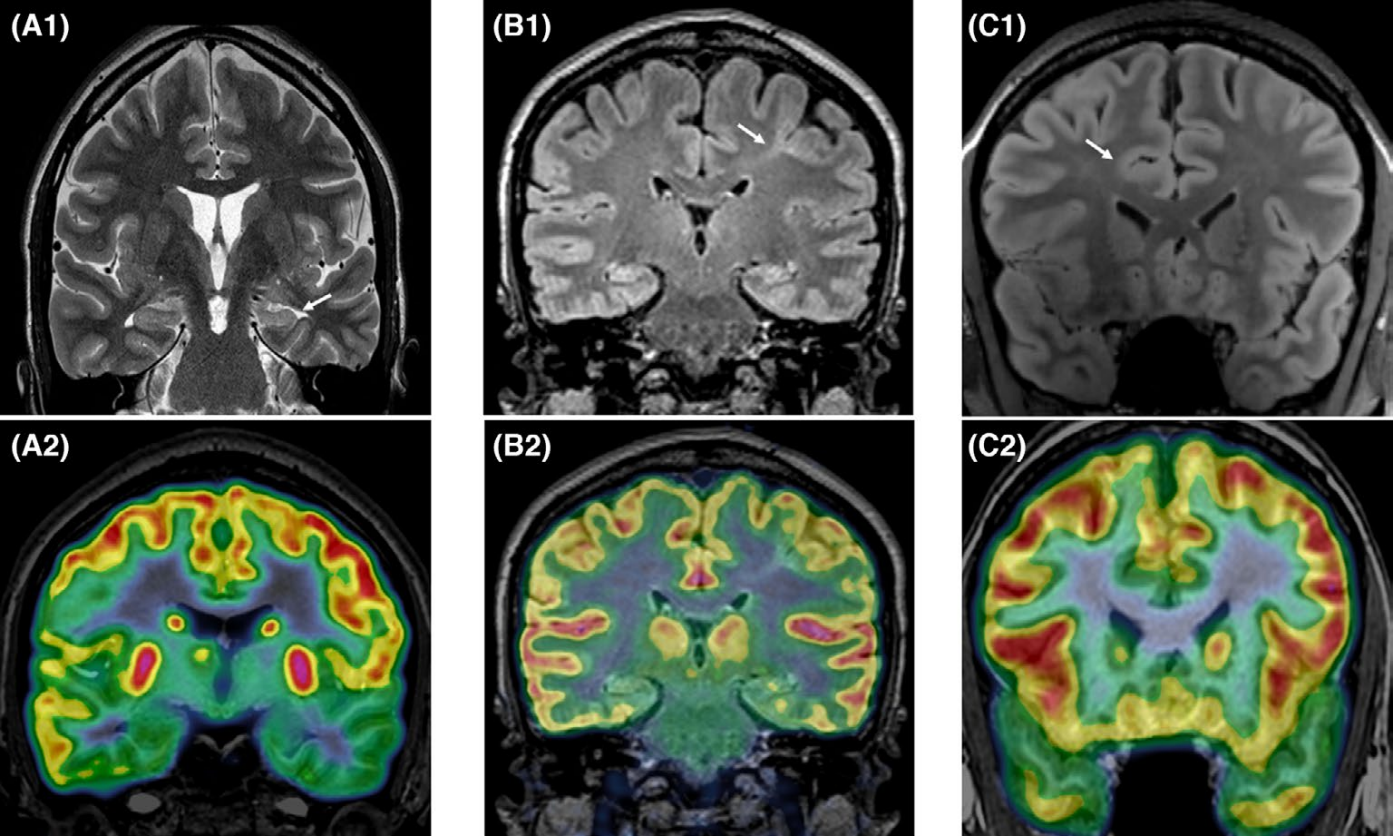
The hypometabolism involving the left temporal lobe on FDG‐PET (a2, fused 3 Tesla MRI/FDG‐PET) in a patient with hippocampal sclerosis (a1, T2‐weighted 3 Tesla MRI, indicated by arrow). Focal bottom of sulcus FDG‐PET hypometabolism (b2, fused 3 Tesla MRI/FDG‐PET) in a patient with focal cortical dysplasia with a typical transmantle sign (b1, FLAIR weighted 3 Tesla MRI) in the left premotor area. Subtle focal cortical dysplasia in the right cingulate gyrus (c1, FLAIR weighted 3 Tesla MRI) with a focal FDG‐PET hypometabolism (c2, fused 3 Tesla MRI/FDG‐PET).

For presurgical assessment, PET imaging commonly relies on the radioactive tracer ^18^[Fluorine]‐fluorodeoxyglucose (FDG), which is injected in the interictal period, typically under EEG surveillance and ideally 24–48 h or more after the last seizure, providing a measure of cerebral glucose metabolism.^79^, ^84^ In people with focal epilepsy, interictal fluorodeoxyglucose FDG‐PET scans frequently yield focal or more widespread (involving a lobe or hemisphere) hypometabolism (Figure 7).^85^ The clinical significance of FDG‐PET abnormalities is unclear. They may be considered as a marker of the functional deficit zone^13^ or indicate at least partially the epileptogenic zone, thereby contributing to the planning of further presurgical intracranial EEG recordings and neurosurgical resection. A variety of alternative nuclear tracers, [^11^C]MET, [^18^F]fluoroethyltyrosine, and [^11^C]alpha‐methyltryptophan, have been used to better define the metabolic extent of the EZ in various etiologies, and retrospective data suggest a role for [11C]methionine PET to improve postoperative seizure control in low‐grade gliomas.^86^

The data on the predictive value for favorable postsurgical seizure outcome using predominantly FDG‐PET are mixed. In a larger series of MRI‐negative DRE patients, PET hypometabolism was seen in 62% of patients (74% TLE; 56% ETLE).^87^ In TLE, PET concordant with EEG had a comparable seizure‐free outcome as an EEG‐concordant MRI (65% vs. 68%). A recent meta‐analysis supports the notion that a localizing focal FDG‐PET hypometabolism is in favor of a good postsurgical outcome, irrespective of the presence of an epileptogenic lesion on brain MRI, whereas diffuse FDG‐PET hypometabolism is rather associated with a worse seizure outcome following surgery.^88^ In MRI‐negative focal epilepsy, the presence of a circumscribed PET hypometabolism is highly associated with a FCD Type 2, which may explain the better prognosis after surgery.^89^ PET images should be co‐registered to a recent MRI or automatically fused in a PET/MRI system. After an initial visual inspection, quantification or voxel‐based analysis allows comparison with a normal database and may reveal subtle asymmetries or regional hypometabolism. Practice guidelines for PET and SPECT imaging in epilepsy were recently published.^79^

#### 3.2.4 Functional MRI (fMRI)

Identification of eloquent areas is indispensable to mitigate the risk of permanent neurological or cognitive deficits following epilepsy surgery. Eloquent areas are cortical regions that carry or contribute to neurological or cognitive functions that are irreversibly impaired if the area is surgically removed. Functional MRI (fMRI) allows non‐invasive assessment of brain activity by measuring changes in blood oxygen and deoxyhemoglobin levels (blood‐oxygen‐level‐dependent [BOLD] signal) upon localized neuronal activation. For language lateralization, language production (e.g., object naming) and comprehension tasks (e.g., sentence comprehension) are used as stimulation paradigms.^90^ According to the stimulation paradigm, the BOLD signals in four regions of interest are often analyzed, including Broca's (frontotemporal region) and Wernicke's area (temporo‐parietal region). Hemispheric dominance for language representation is usually determined by calculating a laterality index. The great advantage of fMRI is the regional representation of primary motor and sensory functions in addition to distinct language functions (e.g., separate for language production and comprehension), which is fairly well associated with postsurgical language outcomes.^91^

#### 3.2.5 Diffusion tensor imaging (DTI)

**FIGURE 8:**
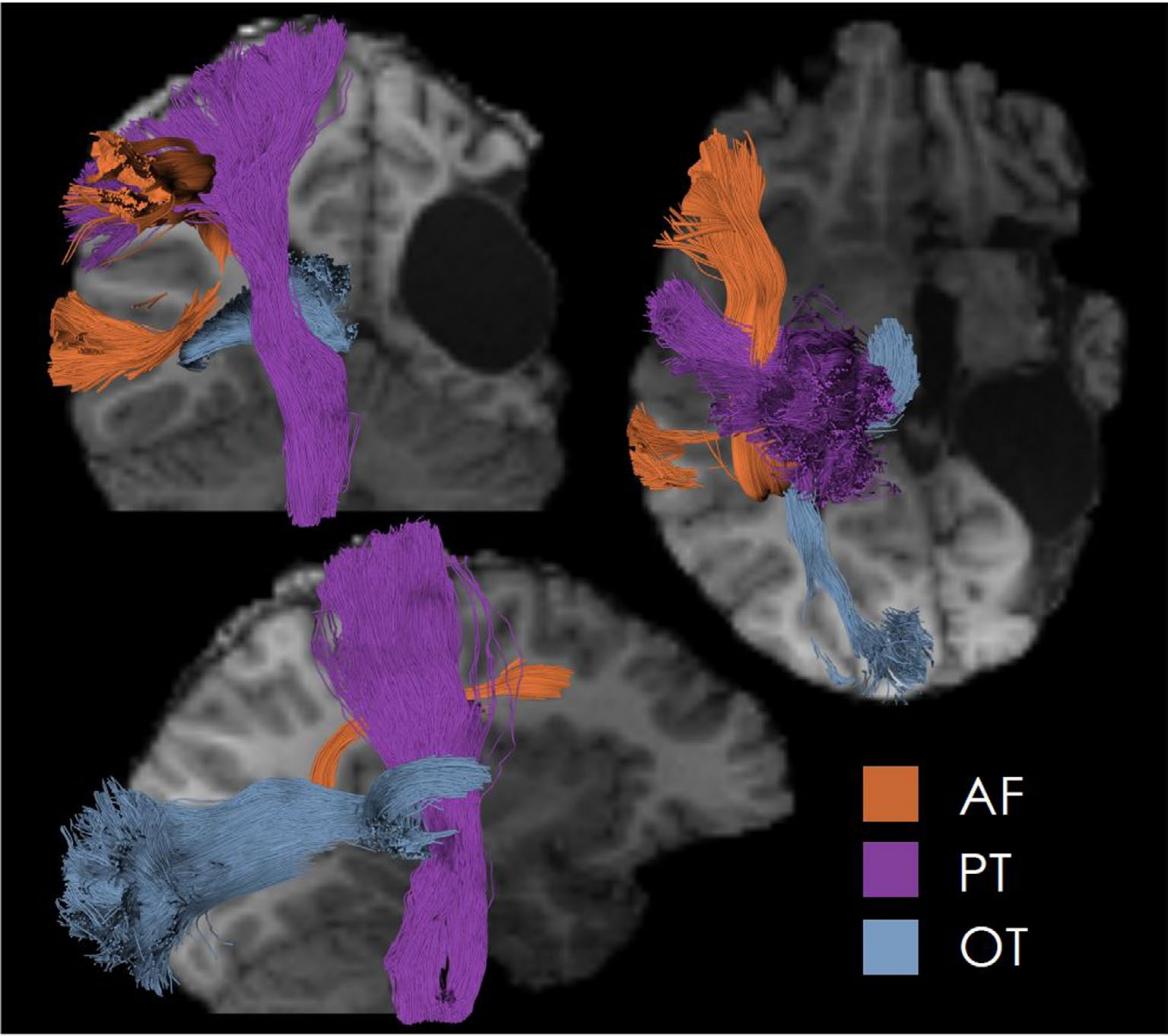
DTI tractography of three essential tracts in presurgical modeling (Gruen et al., 2023) in a 20‐year‐old female patient with porencephaly. AF, arcuate fasciculus; PT, pyramidal tract; OT, optic tract.

DTI and tractography, a modeling technique that uses DTI data, are used to define the course of pathways “hidden” in white matter. Figure 8 illustrates an example of the three most modeled tracts in presurgical evaluation.

Clusters resulting from fMRI can also be used as seed or target masks for DTI tractography.^92^ As a concept rooted in functional anatomy, eloquent areas can only be loosely defined by structural landmarks since they vary between individuals. The results of DTI‐tractography and fMRI can be used to better plan the surgical strategy with the goal of sparing important tracts (e.g., optic radiation, pyramidal tract) and avoiding postsurgical deficits. However, caution is warranted when interpreting the results of DTI tractography and fMRI, as it may be difficult to verify the neuronal underpinnings of virtual fiber bundles.^93^ In addition to functional mapping, DTI has also been used to identify connectivity abnormalities associated with focal epilepsies.^94^

### 3.3 Neuropsychological testing

Comprehensive neuropsychological assessment is an essential part of the multidisciplinary presurgical work‐up, documenting the patient's cognitive strengths and deficits and evaluating emotional and behavioral functioning and psychosocial status.^95^ A neuropsychological evaluation is typically comprised of record review, neurobehavioral examination, and interview with a clinical neuropsychologist followed by performance‐based testing and completion of self‐ or informant‐report questionnaires to assess social, emotional, and behavioral function as well as subjective cognitive concerns. While the specific test measures may vary depending on patient characteristics and clinical context, there is a core set of domains commonly assessed in a presurgical neuropsychological evaluation (Table 4). There is currently no standard clinical neuropsychological battery for epilepsy surgery^96^, ^97^; however, the National Institute of Neurological Disorders and Stroke Common Data Elements for Epilepsy provide some recommendations for neuropsychological instruments to be used in clinical epilepsy research to simplify data sharing and aggregation as well as to facilitate development of evidence‐based guidelines.^98^

**TABLE 4:** Domains commonly assessed in a neuropsychological evaluation with test examples.

Domain	Abilities assessed	Test examples^a^
Intellectual ability	Estimate of premorbid function Verbal intellectual abilities Nonverbal intellectual abilities	Advanced Clinical Solutions Test of Premorbid Function, American National Adult Reading Test, Wide Range Achievement Test—Reading subtest Wechsler Verbal Comprehension Index or verbal subtests Wechsler Perceptual Reasoning Index or nonverbal subtests
Attention	Simple attention Complex attention/working memory Sustained attention	Wechsler Digit Span subtest Wechsler Letter‐Number Sequencing subtest, Wechsler Arithmetic subtest Continuous Performance Test
Processing speed	Speeded mental/visuomotor skills	Wechsler Coding subtest, Wechsler Symbol Search subtest Trail Making Test – Part A
Language	Naming Verbal fluency	Boston Naming Test, Visual Naming Test Phonemic verbal fluency (e.g., Controlled Oral Word Association Test), Semantic verbal fluency (e.g., Animal Fluency)
Executive functioning	Problem‐solving Planning Cognitive flexibility Response inhibition	Wisconsin Card‐Sorting Test DKEFS Tower Test, Tower of London Trail Making Test—Part B DKEFS Color‐Word Interference, Stroop
Visuospatial skills	Visuoperception Visuoconstruction	Judgment of Line Orientation Wechsler Block Design
Episodic memory	Verbal learning/recall/recognition Visual learning/recognition	Rey Auditory Verbal Learning Test, California Verbal Learning Test, Wechsler Logical Memory Wechsler Visual Reproduction, Brief Visual Memory Test
Motor functioning	Motor speed Manual dexterity	Finger Tapping Grooved Pegboard
Performance validity	Stand‐alone and embedded measures of test‐taking effort	Various tests (e.g., Victoria Symptom Validity Scale, Word Memory Test, Advanced Clinical Solutions Word Choice, Reliable Digit Span)
Subjective cognition	Self‐ or caregiver report measures of subjective cognitive abilities (e.g., memory, language)	Memory Assessment Clinics Scale – Epilepsy, Everyday Memory Questionnaire, Everyday Verbal Memory Questionnaire
Social cognition	Theory of mind Social behavior Emotion recognition Empathy Sensitivity to moral and conventional rules	Various tests (e.g., Faux‐Pas Test, Strange Stories Test, Reading the Mind in the Eyes Task, Facial Emotion Recognition, Empathy Questionnaire Social Situation Test, Moral/Conventional Distinction Test)
Emotional/Behavioral function	Self‐ or caregiver‐report screening measures of mood, anxiety, behavior	Various tests (e.g., Beck Depression Inventory, Neurological Disorders Depression Inventory in Epilepsy, Patient Health Questionnaire Depression, Child Depression Inventory, Beck Anxiety Inventory, Patient Health Questionnaire Generalized Anxiety Disorder, Child Behavior Checklist)
Psychosocial status/Function	Self‐ or caregiver report of adaptive functioning	Vineland Adaptive Behavior Scales
Quality of life	Self‐ or caregiver report measures of health‐related quality of life	Quality of Life in Epilepsy, Quality of Life in Childhood Epilepsy

^a^ There is currently no recommended neuropsychological battery for epilepsy. This table lists some of the most commonly used measures, including those recommended as part of the National Institute of Neurological Disorders and Stroke Common Data Elements for Epilepsy, as test exemplars, but is not exhaustive.

The neuropsychologist uses the information gathered during the presurgical assessment to evaluate the patient's cognitive profile in the context of their clinical presentation, other information gathered during the presurgical work‐up (e.g., neuroimaging, electrophysiology), and their medical and psychosocial history. Findings from the preoperative neuropsychological evaluation provide important information regarding how epilepsy, its underlying etiology, and associated factors/comorbidities (e.g., depression/anxiety, sleep problems, psychosocial stressors, medication effects, substance use) have impacted the patient's cognitive and behavioral function and quality of life. Research has demonstrated that approximately 50% of individuals with focal epilepsy have some type of cognitive impairment, with up to 25% of individuals demonstrating impairment in multiple cognitive domains,^99^, ^100^, ^101^ and at least one‐third of people with epilepsy experience a psychiatric disorder over the course of their lifetime.^102^ Importantly, neuropsychological findings can also provide important clues about potential atypical language lateralization. Atypical language lateralization occurs in a large subset (>20%) of patients with focal epilepsy, with the highest rates among those who are left‐handed or ambidextrous.^103^, ^104^ When the pattern of cognitive performance observed on neuropsychological testing does not conform to expected patterns based on known seizure localization or neuroimaging findings, this may suggest atypical language dominance, prompting further assessment with fMRI or another language lateralization procedure.

Preoperative neuropsychological assessment is also essential to aid in the prediction of postoperative cognitive and mood outcomes. Given the elective nature of epilepsy surgery, it is imperative that patients be counseled regarding the potential benefits and risks of the surgical procedure to make an informed decision. While most patients do well from a neuropsychological standpoint following epilepsy surgery, there is a sizeable subset at risk for postoperative declines in cognition or mood. Neuropsychological outcomes following temporal lobe surgeries have been the most well studied, given temporal lobe epilepsy is the most common focal epilepsy and is associated with the highest postoperative cognitive and psychiatric morbidity (i.e., language and memory declines in up to 45% of adults and 25% of children; up to 35% with new or worsening psychiatric symptoms).^105^, ^106^ Numerous risk factors for postoperative neuropsychological decline have been identified over the years, and those most strongly and consistently associated with postoperative outcomes are summarized in Table 5.

**TABLE 5:** Risk factors most strongly associated with negative postoperative neuropsychological outcomes following temporal lobe epilepsy surgery.

Strongest risk factors
Left‐sided (i.e., language‐dominant) surgery
Older age at seizure onset
Older age at surgery
Higher preoperative cognitive ability (e.g., intact memory or naming abilities)
Non‐lesional MRI
Psychiatric history
Relationship status
Seizure severity

The intracarotid amobarbital test, also known as the Wada test, first performed by Juhn Wada, was done over decades to determine the hemispheric lateralization of language function and the risk of postoperative memory impairment.^107^ This procedure is performed by injecting sodium amobarbital (or other anesthetic substances such as methohexital, etomidate, and propofol) into the internal carotid artery in the interictal phase to temporarily inactivate the functions of one hemisphere.^108^, ^109^, ^110^ EEG monitoring allows for the assessment of ipsilateral and diffuse slowing and the rare occurrence of a seizure triggered by the anesthetic.^111^ Wada for memory assessment provides information on the functional reserve of the remaining temporal lobe and, through bilateral injection, the functional adequacy of the epileptogenic lobe.^112^ The memory prediction of the Wada testing is limited given that the hippocampi are vascularized by the posterior circulation. Memory assessment should be part of a multivariate approach, and the role of Wada is nowadays limited to select patients.^113^, ^114^

It can be quite difficult to predict postoperative neuropsychological outcomes, particularly in those individuals with conflicting risk (e.g., older age at surgery, intact memory) and resilience (e.g., early age at onset, mesial temporal sclerosis on MRI) factors. To address this issue, recent multicenter studies have developed and validated multivariate models and nomograms that make use of preoperative variables easily accessible at most epilepsy centers to estimate the probability of postoperative cognitive or mood decline. These models have very good predictive accuracy in identifying patients at high risk for cognitive or mood declines and are publicly available for clinical use (Adult Nomograms: https://riskcalc.org/CognitiveAndMoodAfterEpilepsySurgery/; Pediatric Nomograms: https://riskcalc.org/MemoryAfterPediatricEpilepsySurgery/).^115^, ^116^, ^117^, ^118^ While this type of predictive modeling work is still in its infancy, efforts to improve prediction of postsurgical neuropsychological outcomes by including more sophisticated predictor variables (e.g., neuroimaging and EEG features) are ongoing.^119^, ^120^ Nevertheless, clinical data collected during the preoperative work‐up, including results of comprehensive neuropsychological testing, are essential to aid the neuropsychologist in making recommendations to improve patient functioning and quality of life, estimating a given patient's risk for postoperative cognitive or mood decline, and providing appropriate presurgical counseling. Neuropsychologists are integral members of the multidisciplinary epilepsy surgery team and routinely discuss results of the preoperative neuropsychological evaluation and any associated recommendations during the patient management conference to aid clinical decision‐making.

Postoperative neuropsychological assessment is an important part of clinical care. Repeat neuropsychological assessment is typically recommended 6–12 months following epilepsy surgery to assess any changes in cognitive, emotional, behavioral, or social functioning and to guide postoperative recommendations, including potential treatment targets and resources (e.g., cognitive rehabilitation, speech‐language therapy, psychotherapy, school/work accommodations).

### 3.4 Planning and implantation of intracranial electrodes

#### 3.4.1 Candidate selection and planning

In general, an invasive implantation is indicated in patients where phase 1 presurgical evaluation does not allow proceeding to direct surgery as non‐invasive data are discordant or insufficient, but only if a hypothesis of a single and potentially operable seizure focus is supported. In the last two decades, the majority of invasive EEG implantations are performed using a stereotactic approach with intracranial depth electrodes.^121^ The ability of SEEG to provide stereotactic targeting of the epileptic network benefits from electrical and magnetic source imaging of the interictal and ictal activity when planning electrode trajectories.^14^, ^122^, ^123^ Stereoelectroencephalography (SEEG) has a reduced morbidity and mortality rate and favorable seizure outcome compared with a craniotomy‐based subdural grid evaluation according to larger surgical series and a comparative effectiveness analysis.^124^, ^125^ However, a subdural grid evaluations provide better coverage over specific brain areas such as the lateral convexity, basal temporal area, and arguably the interhemispheric cortex and offer more comprehensive electrical stimulation mapping close to eloquent cortex.^126^

**FIGURE 9:**
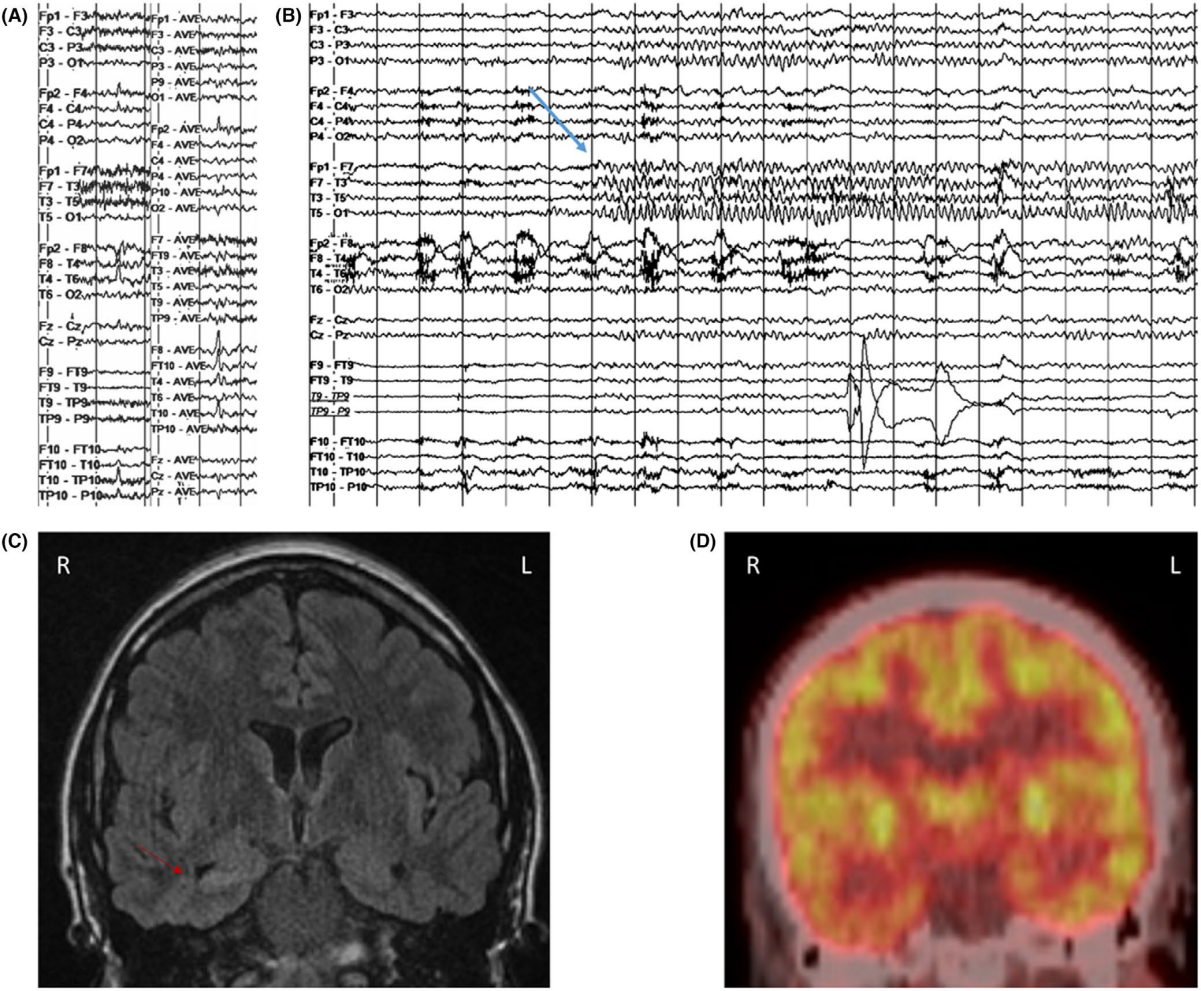
Phase I work‐up in a 27‐year‐old right‐handed male with a family history of seizures in his maternal grandmother. Epilepsy onset was at age 19 years with focal impaired consciousness seizures occurring multiple times per month despite multiple antiseizure medications. Seizures were recorded as episodes of behavioral arrest with orolingual automatisms characterized by lip smacking followed by postictal fatigue. He did not have postictal aphasia. (A) Interictal EEG showed frequent right anterior to mid‐temporal spikes (maximum negativity over F8 > FT10 > T10) (1–70 Hz bandpass, bipolar longitudinal montage with subtemporal chains). Clinical onset (aura) preceded electrographic onset, with (B) first ictal changes on scalp EEG characterized by rhythmic sinusoidal 5–6 Hz theta activity over the left hemisphere maximal over the left temporal region (arrow) (20s, 1–70 Hz bandpass, bipolar longitudinal montage with subtemporal chains). (C) 3 T Brain MRI showed reduced right hippocampal volume with hyperintense T2 signal consistent with right mesial temporal sclerosis, and (D) FDG‐PET showed right temporal hypometabolism. Neuropsychometric testing was largely normal with preserved left mesial temporal function. In summary, there was a discrepancy between scalp EEG seizure onset in the left temporal region and a lesion, irritative zone (interictal discharges), and functional deficit zone (FDG‐PET) in the right mesial temporal lobe. Additionally, seizure semiology suggested a symptomatogenic zone within the mesial temporal lobe in the absence of definitive lateralizing features, and the patient did not have a focal neuropsychological deficit. As a result, a phase II evaluation with stereoelectroencephalography (SEEG) monitoring was recommended to test the hypothesis for the epileptogenic zone (Figure 13). His 5‐SENSE score suggested that SEEG is likely to identify a focal seizure onset zone. Computation of his Specific Consistency Score suggests a score of 6 (grade B) which indicates that his data were partially consistent and further supports the decision for invasive monitoring.

**FIGURE 10:**
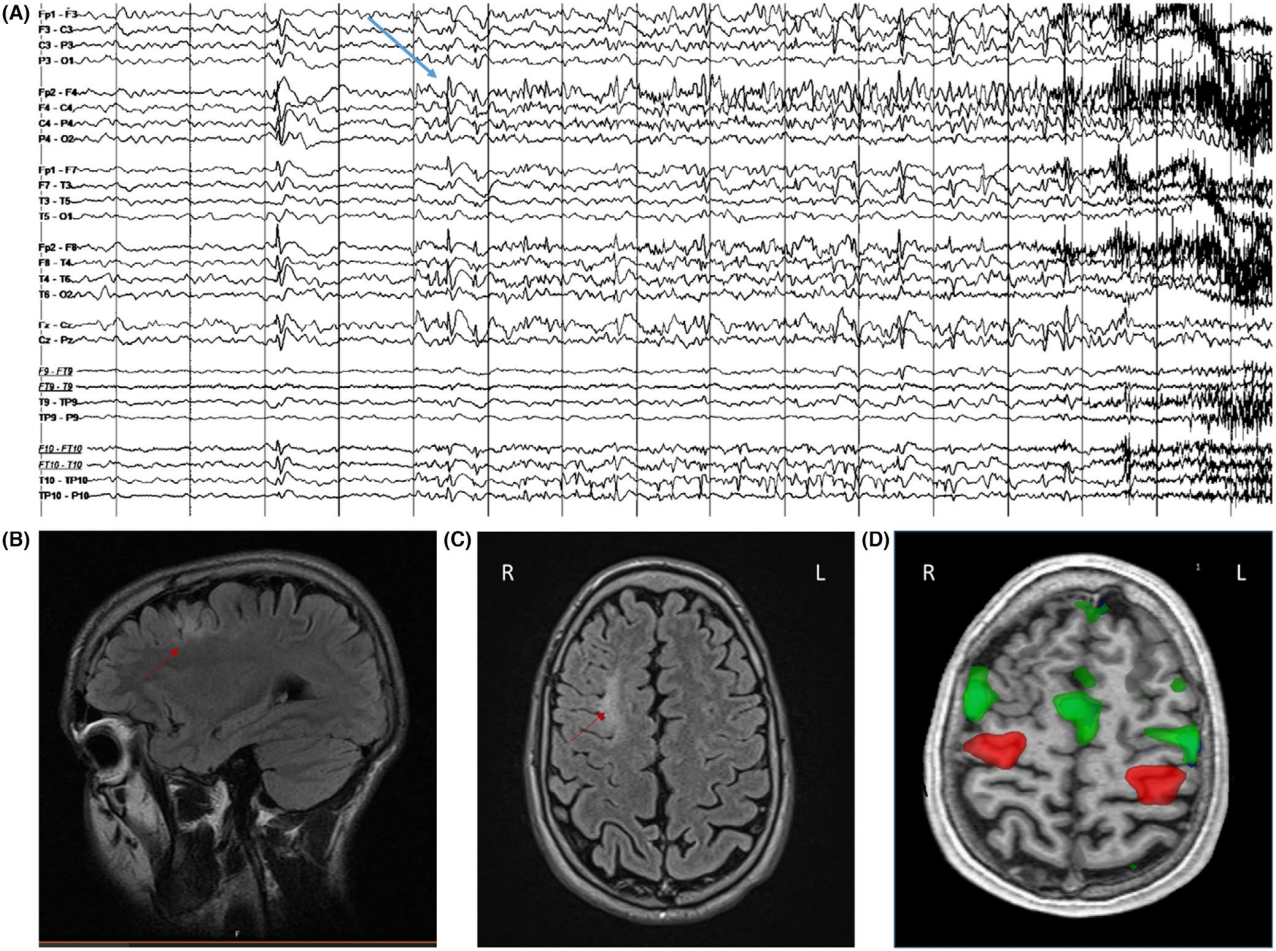
Phase I work‐up in a 24‐year‐old right‐handed male with a family history of epilepsy in his paternal uncle and nephew. Epilepsy onset was at age 7 years, initially with focal impaired consciousness seizures during childhood characterized by left‐sided facial twitching and leftward gaze deviation which no longer occurred after the age of 9. Subsequently, all seizures were focal to bilateral tonic–clonic, occasionally preceded by an ill‐defined aura described as full body discomfort, occurring monthly. Seizures were recorded as episodes typically occurring out of sleep and rarely preceded by an ill‐defined aura (patient alerts observer or pushes the alarm, however, is unable to explain his symptoms) and hyperventilation, with subsequent versive head turn to the left, gaze deviation to the left, tonic posturing with Figure 4 (flexion of the right upper extremity, extension of the left upper extremity), followed by bilateral clonic activity with postictal confusion. (A) Clinical onset typically occurred 8–10 s after electrographic onset, with first ictal changes on scalp EEG characterized by spike and slow wave discharges followed by 10–12 Hz repetitive spiking over the right frontal region (with maximum negativity over F4 > Fp2 > Fz) (arrow, 16 s, 1–70 Hz bandpass, bipolar longitudinal montage with subtemporal chains). (B, C) 3 T Brain MRI showed T2/FLAIR hyperintense signal and blurring of the gray‐white matter junction at the level of right superior frontal sulcus. (D) Functional MRI showed left dominant expressive and receptive speech with accessory expressive speech within the right frontal lobe 1–2 cm inferior and lateral to the lesion (green), and left hand motor function within 1 cm posterior and lateral to the right frontal lobe lesion (red). Neuropsychological evaluation results were not lateralizing or localizing. The primary hypothesis for the epileptogenic zone was a right frontal focus based on the scalp EEG seizure onset pattern, lesion within the right superior frontal lobe, and early elementary motor manifestations. Intracranial EEG monitoring was recommended to identify margins for potential surgical resection and for motor mapping given proximity of the lesion to the hand motor region (Figure 14). His 5‐SENSE score suggested that SEEG is likely to identify a focal seizure onset zone. Computation of his Specific Consistency Score suggests a score of 7 (grade B) which indicates that his data were partially consistent and further supports the decision for invasive monitoring.

**FIGURE 11:**
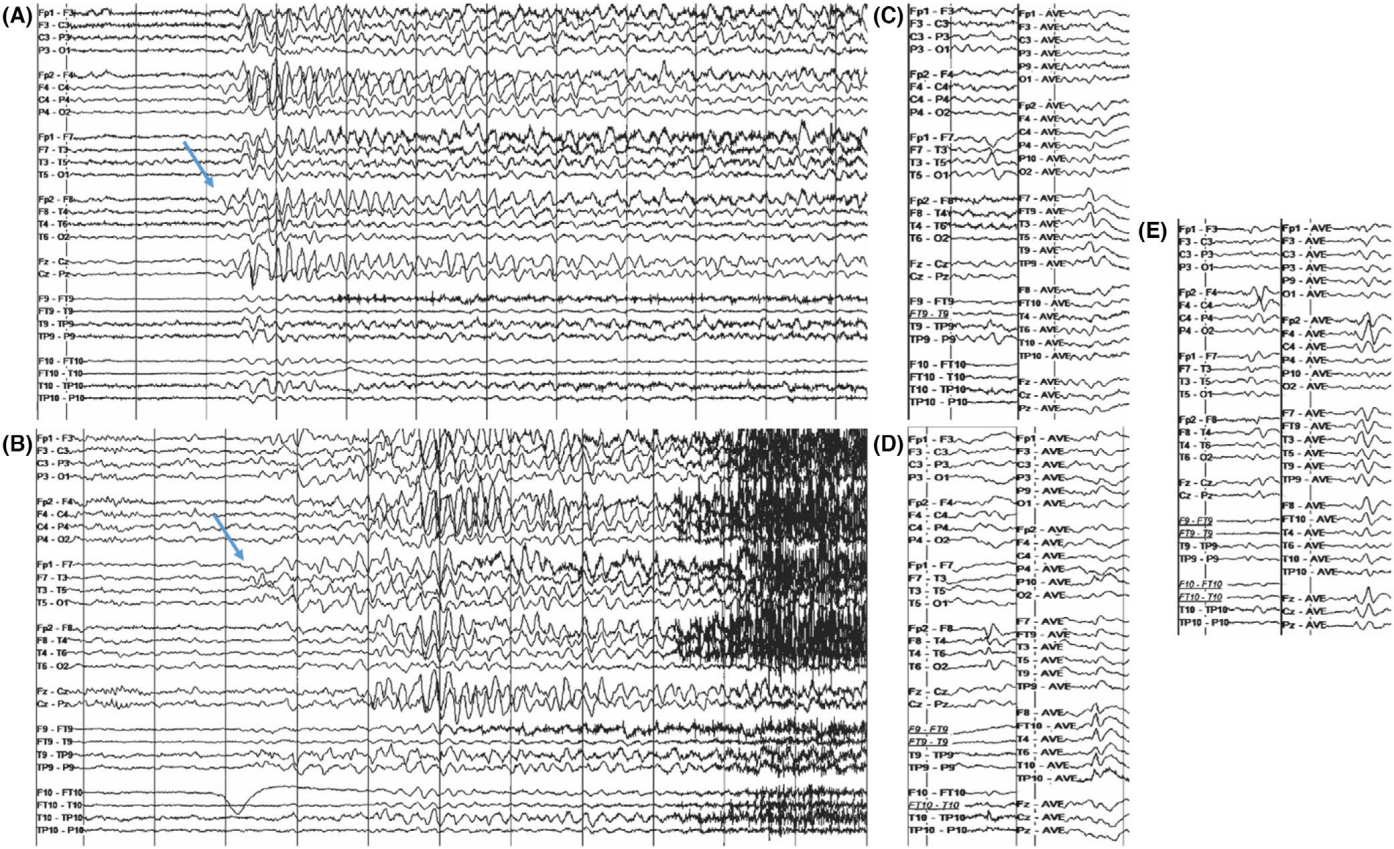
Phase I work‐up in a 56‐year‐old left‐handed woman with no known seizure risk factors. Epilepsy onset was at age 49 years with focal impaired consciousness seizures occurring multiple times per month and focal to bilateral tonic–clonic seizures occurring 2–3 times per year. Seizures were recorded as episodes of slow slightly asymmetric tonic posturing of the L > R upper extremities characterized by elevation of the arms with flexion at the elbows followed by brief clonic jerks, and decreased responsiveness with eyes closed, in the absence of a preceding aura. In the postictal phase, she was confused but able to communicate. The first ictal changes on scalp EEG were characterized by bilateral synchronous ictal onset with diffuse rhythmic 4 Hz theta activity, occasionally with a unilateral lead‐in alternating between the right (A) and left (B) frontotemporal regions (arrows) (12 s, 1–70 Hz bandpass, bipolar longitudinal montage with subtemporal chains). The interictal EEG showed multifocal interictal discharges over the left anterior to mid‐temporal region (with maximum negativity over FT9 > T3) (C), the right anterior to mid‐temporal region (maximum negativity over FT10 > T4) (D), and over the right mid‐frontal region (maximum negativity over F4 > Fz) (E). 3T Brain MRI was non‐lesional (not shown in Figure). Neuropsychometric testing revealed deficits on measures of executive functioning and complex visuospatial organization, with below‐average visual memory function, overall suggestive of right frontal and non‐dominant parietal/posterior quadrant dysfunction. As the patient was left‐handed, a functional MRI for evaluation of language function was obtained (not shown here) and showed right hemispheric expressive and receptive speech. In summary, her overall data were not concordant, with a poorly lateralizing semiology, rapid bilateral synchronous ictal onset on scalp EEG, multifocal interictal discharges, non‐lesional brain MRI, and neuropsychological evaluation suggestive of multi‐lobar dysfunction. As such, the likelihood of identifying a focal resectable generator was estimated to be low. Her Specific Consistency Score was 2 (Grade C) suggestive of inconsistent data for surgical resection, and her 5‐Sense Score was 19.8, indicative of a non‐focal epilepsy. The patient was offered neuromodulation with deep brain stimulation of the centro‐median nucleus or with vagal nerve stimulation, and she opted for the latter. Seizure frequency has improved, though she continues to have monthly focal impaired consciousness seizures at her 1‐year follow‐up appointment.

**FIGURE 12:**
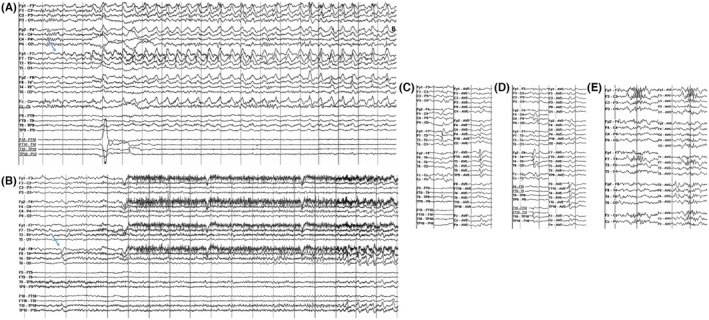
Phase I work‐up in a 40‐year‐old left‐handed woman with no known seizure risk factors. Epilepsy onset was at age 24 years, with focal impaired consciousness seizures occurring 1–2 times per month, in addition to monthly focal to bilateral tonic–clonic seizures. Seizures were recorded as episodes of staring, orolingual automatisms, rarely with dystonic posturing of the left upper extremity, and confused behavior in the absence of an aura. In the postictal phase, she was able to communicate. Ictal onset on scalp EEG was characterized by rhythmic spike and slow wave discharges over the left frontotemporal region (A) or by a broad sharp over the right anterior temporal and basal temporal region, followed by rhythmic theta activity and then by low voltage fast activity, with subsequent evolution consisting of rhythmic spike and slow wave discharges over the right anterior temporal region (B) (18 s, 1‐70 Hz bandpass, bipolar longitudinal montage with subtemporal chains). The interictal EEG showed multifocal interictal discharges over the left anterior to mid‐temporal region (with maximum negativity over FT9 > F7 > T3) (C), the right anterior to mid‐temporal region (maximum negativity over FT10 > T4) (D), and over the left frontal region (F3 > F7 > Fp1) (E). 3T brain MRI was non‐lesional (not shown in Figure). Interictal FDG‐PET scan did not reveal areas of focal hypometabolism and was non‐diagnostic (not shown in Figure). Neuropsychometric testing revealed mild non‐lateralizing frontotemporal/anterior temporal weakness. In summary, her overall data were suggestive of a multifocal epilepsy, and the likelihood of identifying a focal resectable EZ was estimated to be low. Her Specific Consistency Score was 3 (Grade C), suggestive of inconsistent data for surgical resection, and her 5‐Sense Score was indicative of non‐focal epilepsy. Deep brain stimulation of the anterior nucleus of the thalamus was pursued, resulting in partial improvement in seizure frequency.

The most frequent unwanted outcome of invasive monitoring is that patients undergoing the procedure can subsequently not be offered resective surgery. For SEEG, this percentage ranges from 20% at experienced centers to 45% after bihemispheric implantations.^127^ Appropriate candidate selection is key to minimizing this number. Some non‐resectable patients may be offered neurostimulation; however, closed or open loop neurostimulation on its own is not an indication for an invasive evaluation. An invasive evaluation should only be offered on the basis of one strong hypothesis and at least 1 and a maximum of 2 alternative hypotheses. Implantations without a clear hypothesis of a potentially resectable EZ are to be avoided given that a typical SEEG exploration with 192 ± 54 contacts covers only about 5–10% of brain volume.^128^ A useful tool in deciding on a SEEG implantation is the 5‐SENSE score which predicts the likelihood of detecting a focal seizure onset zone when going ahead with SEEG.^129^ It is a simple algorithm integrating information from the basic modalities of a phase 1 evaluation consisting of semiology, ictal and interictal scalp EEG, neuroimaging, and neuropsychological testing. A good indication for detecting a focal seizure onset zone in SEEG is the presence of a focal MRI lesion, regional ictal EEG onset extent, absence of independent bilateral interictal epileptiform anomalies (with the exception of bitemporal IEDs), a strong localizing semiology, as well as a focal neuropsychological deficit (https://lab‐frauscher.github.io/Sense_calc/). Figures 2 and 9, 10, 11, 12 provide case examples to illustrate where SEEG is not indicated as phase 1 work‐up allows for proceeding to direct surgery (Figure 2), where SEEG is indeed useful (Figures 9, 10), and where SEEG should not be pursued as information is not pointing to a strong hypothesis of a single focal generator (Figures 11, 12).

**FIGURE 13:**
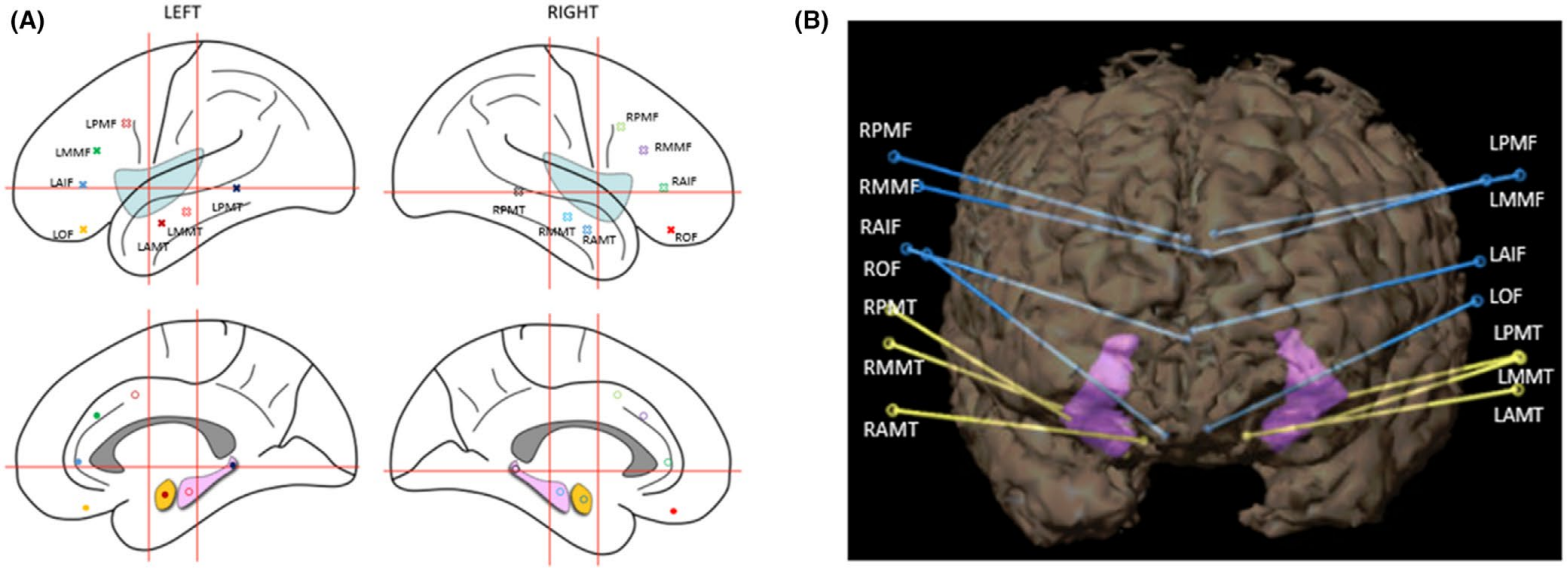
The implantation strategy of the patient discussed in Figure 9 queried a right mesial temporal primary hypothesis and left mesial temporal versus bilateral mesial temporal secondary hypotheses. This was due to the discrepancy between left temporal seizure onset on scalp EEG and a right temporal irritative zone, functional deficit zone, and lesion, in the absence of a localizing neuropsychological deficit or of lateralizing semiological features. Implantation scheme (A) and trajectories (B) with 14 electrodes sampling the bilateral temporal, orbitofrontal, and cingulate cortex. The first letter specifies laterality: R (right) or L (left); the second letter specifies the anterior–posterior position: A (anterior), M (middle), or P (posterior); the third letter specifies the superior–inferior position: S (superior), M (middle), or I (inferior). the fourth letter specifies the lobe: F (frontal), T (temporal), P (parietal), or O (occipital). OF indicates the orbitofrontal region. Example, “RAMT” refers to the right anterior middle temporal region.

**FIGURE 14:**
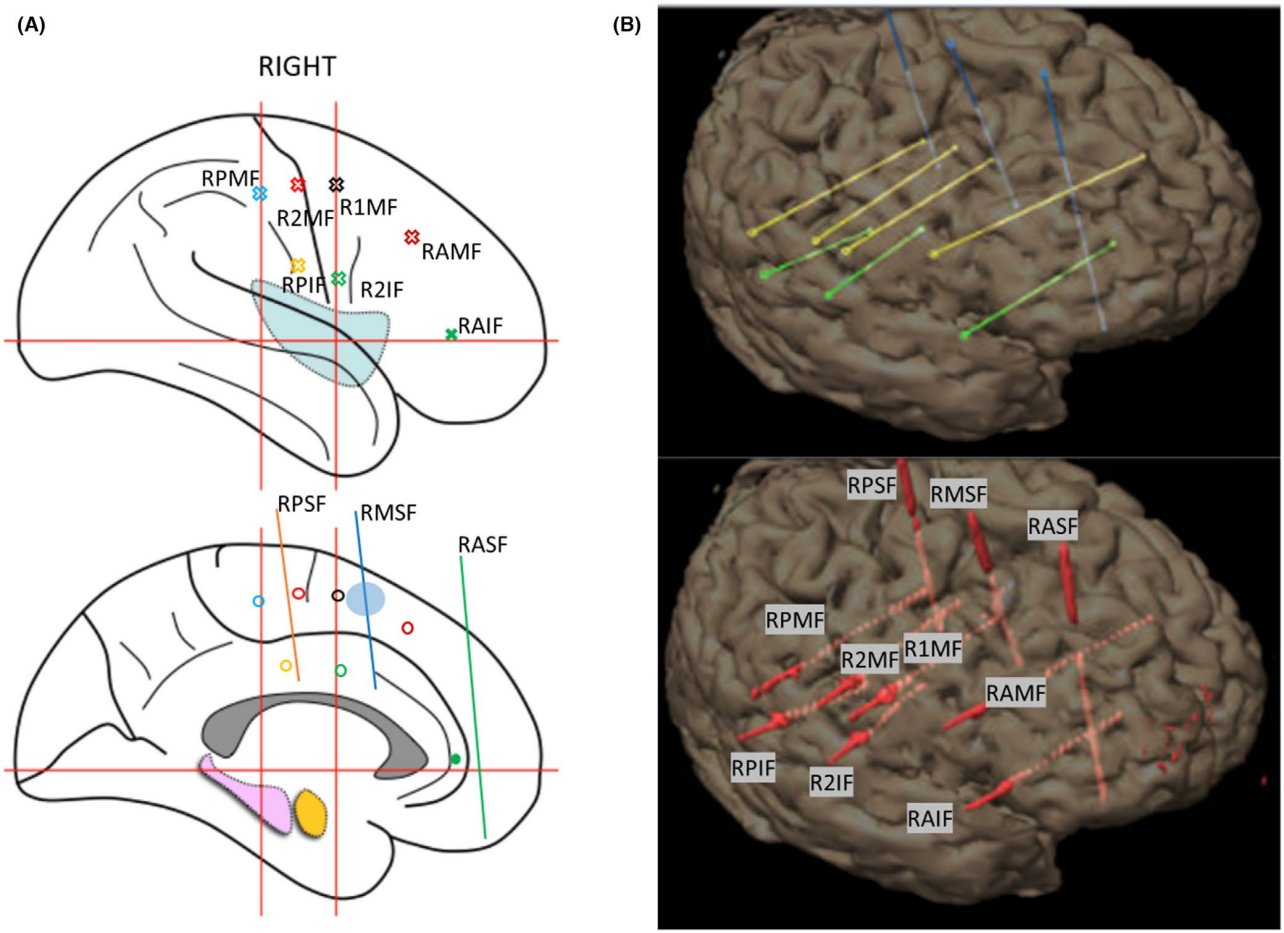
The implantation strategy of the patient discussed in Figure 10 queried a right superior frontal lesional epilepsy as the primary hypothesis, and the electrode configuration (A) targeted the lesion and its anterior, inferior, and posterior margins, including the motor cortex, in order to identify margins for potential surgical resection and for motor mapping. The reconstructed trajectories on the Brainlab software are shown in (B). The first letter specifies laterality: R (right) or L (left); the second letter specifies the anterior–posterior position: A (anterior), M (middle), or P (posterior); the third letter specifies the superior–inferior position: S (superior), M (middle), or I (inferior); the fourth letter specifies the lobe: F (frontal), T (temporal), P (parietal), or O (occipital). OF indicates the orbitofrontal region. For example, “RASF” refers to the right anterior superior frontal region.

Figures 13 and 14 provide further information on the planning strategy to allow exploration of the main hypothesis, 1 alternative hypothesis as well as to delineate resection margins.

#### 3.4.2 Synopsis of interpretation

**FIGURE 15:**
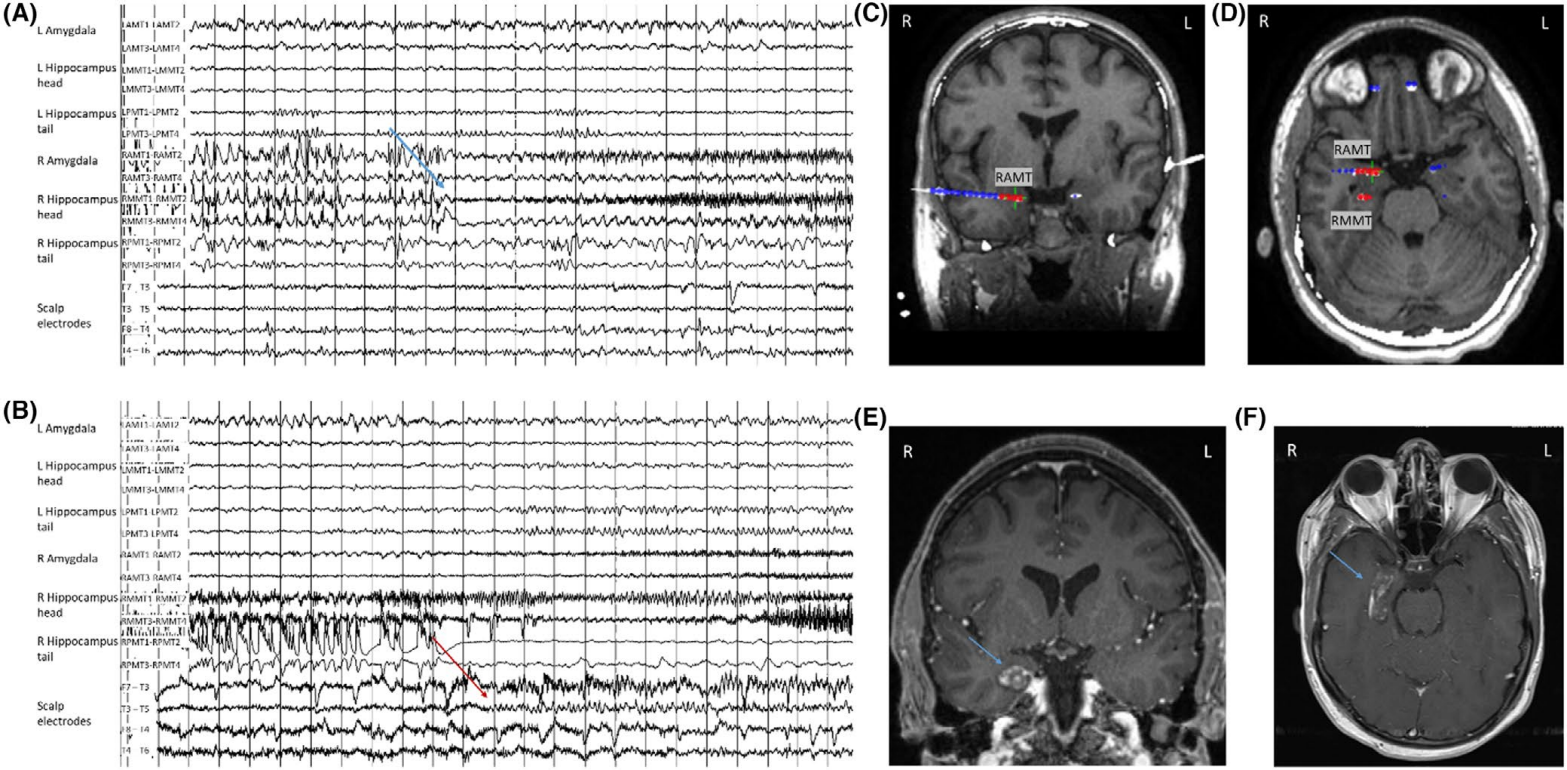
Ictal SEEG (A, B), reconstructed electrodes (C, D), and surgical outcome (E, F) of the patient discussed in Figures 9 and 13. The SEEG page shown in A (25 s, LFF 1 Hz, HFF off, modified bipolar montage with selected channels for clarity) demonstrates pre‐ictal repetitive spiking followed by ictal onset characterized by low voltage fast activity (arrow) over the hippocampal head (RMMT 1–2) closely followed by right amygdala (RAMT 1–2). The page shown in B demonstrates scalp EEG onset characterized by rhythmic 6 Hz theta activity over the left temporal region, which is noted 70 s after SEEG ictal onset, and correlates with ictal propagation to the left hippocampus on SEEG (LPMT) characterized by rhythmic 6‐7 Hz theta activity. Electrodes were visualized by image co‐registration between a pre‐implantation T1‐weighted volumetric MRI and a post‐implantation volumetric CT using the Brainlab software and Curry 8 software, with most active contacts at seizure onset highlighted in red (C, D). These findings confirmed a right mesial temporal lobe epilepsy and suggested that the seizures seen on scalp EEG were falsely lateralizing. The patient opted for right mesial temporal laser interstitial thermal therapy (LiTT) and the ablation zone is shown in E and F. He remains seizure‐free with an Engel IA outcome at 5 years. The first letter specifies laterality: R (right) or L (left); the second letter specifies the anterior–posterior position: A (anterior), M (middle), or P (posterior); the third letter specifies the superior–inferior position: S (superior), M (middle), or I (inferior); the fourth letter specifies the lobe: F (frontal), T (temporal), P (parietal), or O (occipital). OF indicates the orbitofrontal region. Example, “RAMT” refers to the right anterior middle temporal region.

**FIGURE 16:**
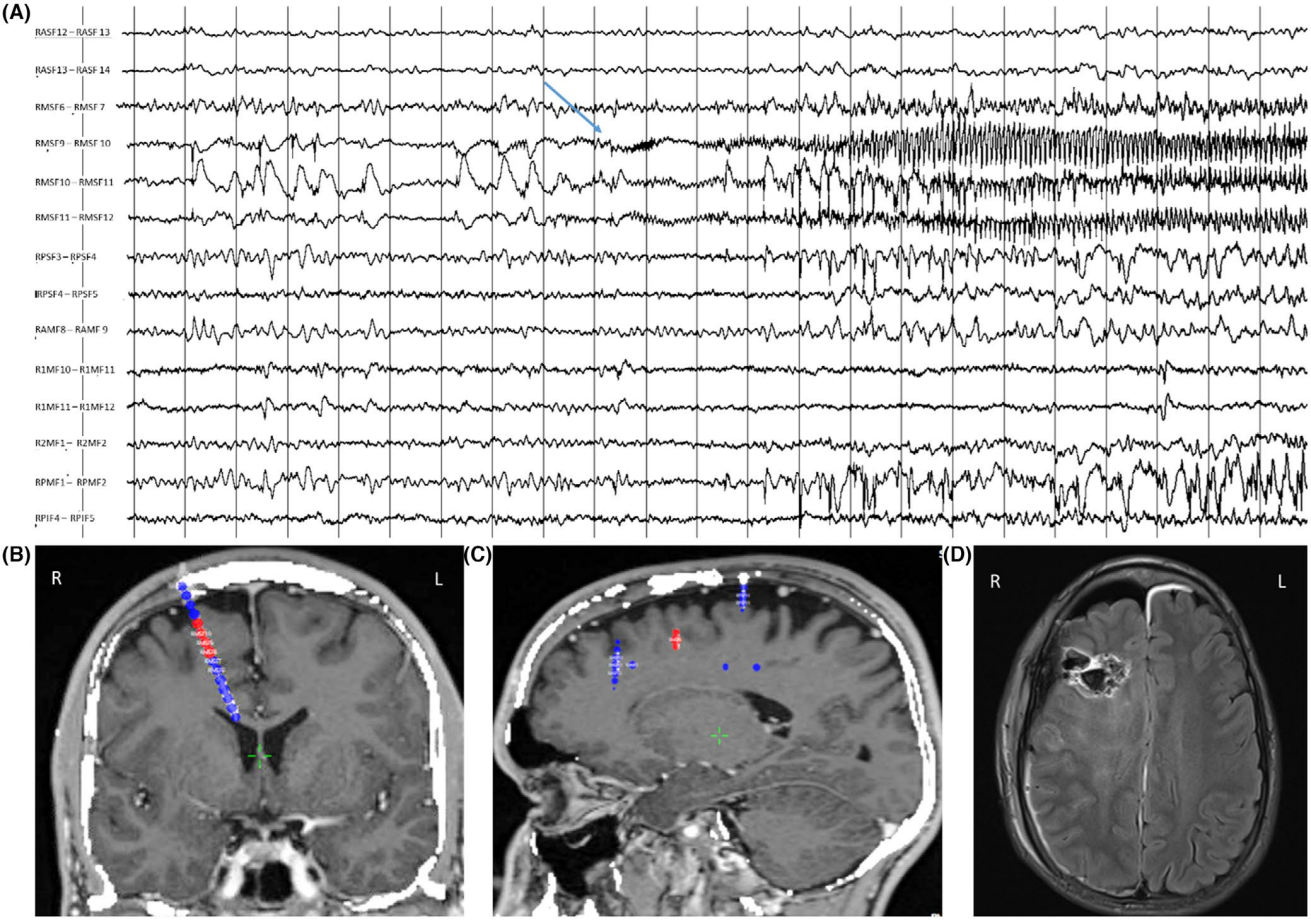
Ictal SEEG (A), reconstructed electrodes (B, C), and surgical outcome (D) of the patient discussed in Figures 10 and 14. The SEEG page shown in A (25 s, LFF 0.3 Hz, HFF off, modified bipolar montage with selected channels for clarity) demonstrates pre‐ictal spiking followed by focal ictal onset characterized by low voltage fast activity (arrow) followed by rapid repetitive spiking over the right superior frontal gyrus (contacts RMSF 9–10 > RMSF 11–12 within lesion). Electrodes were visualized by image co‐registration between a pre‐implantation T1‐weighted volumetric MRI and a post‐implantation volumetric CT using the Brainlab software and Curry 8 software, with most active contacts at seizure onset highlighted in red (B, C). Functional cortical mapping suggested the absence of eloquent motor within the lesion (with stimulation of RMSF) and helped identify the margins for surgical resection (RPSF, R1MF, R2IF, and R2MF within motor cortex). A right frontal lesionectomy was performed (D), and microscopic examination of the pathologic specimen revealed dysmorphic neurons with disorganized neuronal arrangements in the absence of balloon cells, consistent with a focal cortical dysplasia type IIa. The patient remains seizure‐free with an Engel IA outcome at 6 years. The first letter specifies laterality: R (right) or L (left); the second letter specifies the anterior–posterior position: A (anterior), M (middle), or P (posterior); the third letter specifies the superior–inferior position: S (superior), M (middle), or I (inferior); the fourth letter specifies the lobe: F (frontal), T (temporal), P (parietal), or O (occipital). OF indicates the orbitofrontal region. *Example, “RASF” refers to the right anterior superior frontal region*.

Interpretation of seizures in surface and intracranial EEG follows the important principle of ictal anatomo‐electroclinical correlation. This is complemented by investigation of the background EEG, the interictal EEG, and electrical stimulation with the purpose of functional mapping and seizure stimulation.^3^ Figures 15 and 16 provide a synopsis of the main SEEG results of the above 2 patients. Importantly, intracranial EEG goes along with an inherent sampling limitation. This implies that the apparent SOZ is not necessarily the “true” SOZ. Red flags are clinical symptoms prior to first EEG changes, no interictal epileptiform activity in that area, absence of a build‐up of spikes prior to seizures, as well as absence of seizure onset patterns without low voltage fast activity. However, low voltage fast activity is also seen in propagation areas and is therefore alone not a good indicator of appropriate localization of the SOZ.

#### 3.4.3 Role of intraoperative electrocorticography during resection

Intraoperative electrocorticography (ioECoG) has been practiced in epilepsy surgery centers around the world for many decades, with its yield being debated more recently and continued to be pursued in only a modest number of centers. IoECoG offers neurosurgeons instantaneous readouts of pathologic EEG activity. It may help to delineate the irritative zone, especially in focal and superficial lesions, improve the reliability of functional mapping through monitoring for after‐discharges and seizures, and identify residual IEDs post‐resection. A recent meta‐analysis comprising 3631 patients with ioECoG‐tailored surgery showed that ioECoG‐tailored surgeries reached a higher rate of favorable seizure outcomes than non‐ioECoG‐tailored surgeries. This evidence was highest for tumors as well as for complete removal of IED areas.^130^ These results may inspire the use of ioECoG more widely, including in tumor‐associated epilepsy.^131^

## 4 ETIOLOGIES AMENABLE TO SURGICAL TREATMENT AND THEIR PROGNOSES

**FIGURE 17:**
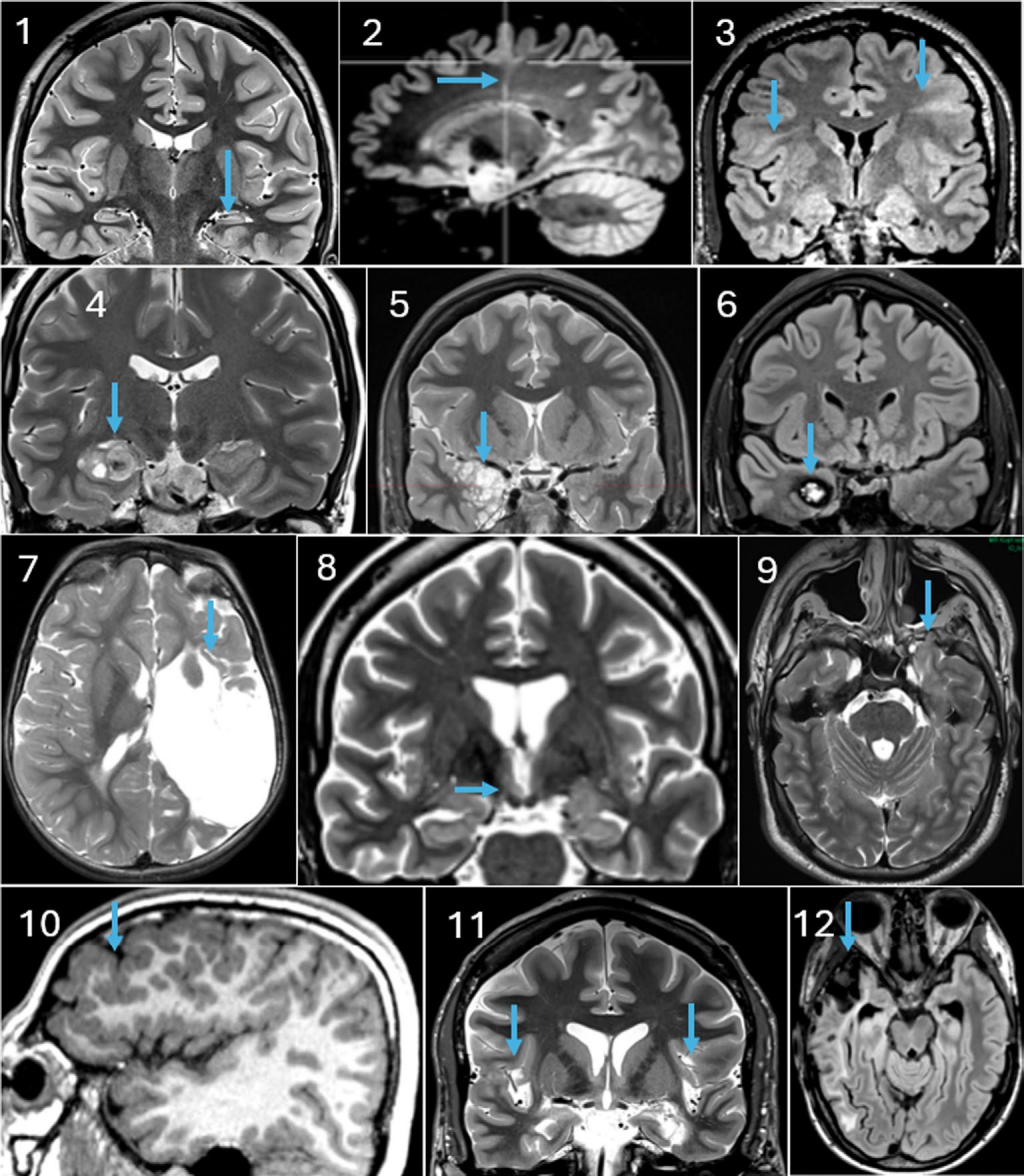
MR imaging gallery of common etiologies underlying focal epilepsy. 1. Hippocampal sclerosis, 2. FCD IIB with transmantle sign; 3. TSC with multiple tubers left and right frontal, 4. Ganglioglioma with cystic and calcified components, 5. DNET (multivacuolar), 6. Cavernoma, 7. Postischemic defect in the territory of the left MCA, 8. Hypothalamic hamartoma type II, 9. Encephalocele left temporal, 10. Frontal polymicrogyria, 11. Postencephalitic defects temporo‐insular, 12. Postcontusional defects right temporal.

Whereas early on mesial temporal lobe epilepsy due to hippocampal sclerosis was the most common target for epilepsy surgery, this has changed considerably with progress in high‐resolution MR imaging^132^ and with increasing rates of children undergoing epilepsy surgery.^133^ Meanwhile, the spectrum of etiologies has increased considerably, and in larger surgical programs, malformations of cortical development in their various forms, and particularly focal cortical dysplasia, have become the most common underlying brain pathology not only in childhood but at all ages (Figure 17); hippocampal sclerosis, long‐term epilepsy‐associated tumor (LEAT), cavernomas, and gliosis follow with lower incidence.^134^

**FIGURE 18:**
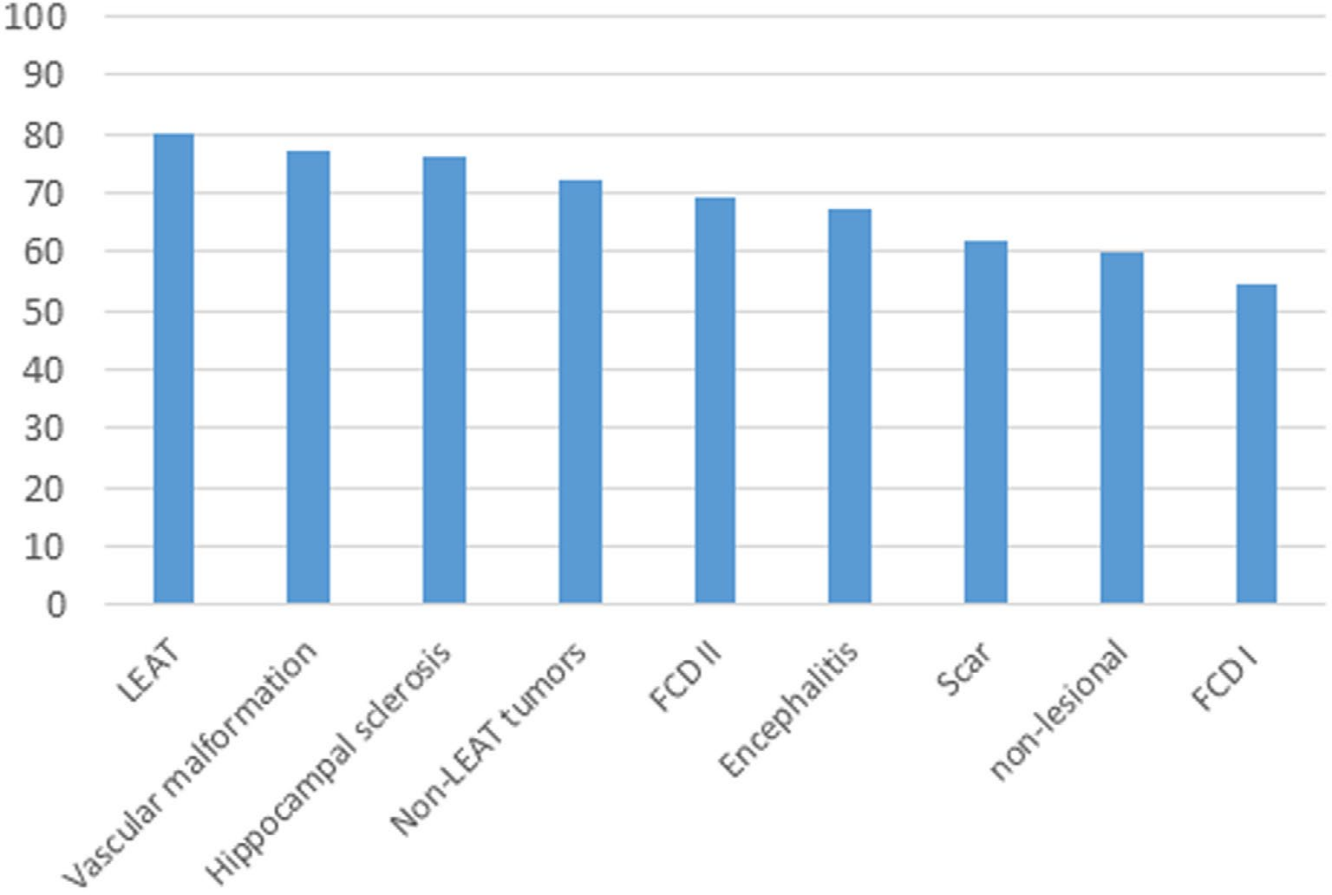
Etiology‐dependent outcomes based on 3273 patients from a histological database.^26^

The etiology of structural epilepsy is a key determinant of postoperative seizure freedom following epilepsy surgery, and patients without identified etiology perform worst in terms of epilepsy surgery outcome.^135^ In the largest multicenter series reported,^26^ two‐year postoperative seizure freedom was most frequently achieved with resections of LEATs,^136^ followed by vascular lesions and hippocampal sclerosis (Figure 18). Worthwhile but less favorable postoperative outcomes following surgery have repeatedly been shown in patients with hemimegalencephaly.^35^, ^137^, ^138^, ^139^ Despite their high prevalence, some structural epilepsies require diligent selection of suitable candidates, including polymicrogyria or encephalomalacia from a remote vascular or traumatic scar, which show reasonable seizure improvement in well‐selected candidates.

In several etiologies, the duration of epilepsy additionally affects long‐term outcomes; this was early reported in mesial temporal epilepsy due to hippocampal sclerosis^140^ and in FCD^141^, ^142^ but was recently shown also in other etiologies.^26^ Secondary epileptogenesis due to kindling‐like phenomena (e.g., in the contralateral hippocampus^143^) or changes in network excitability beyond the primary lesion^144^ have been discussed as additional etiology‐dependent mechanisms of secondary epileptogenesis.

**FIGURE 19:**
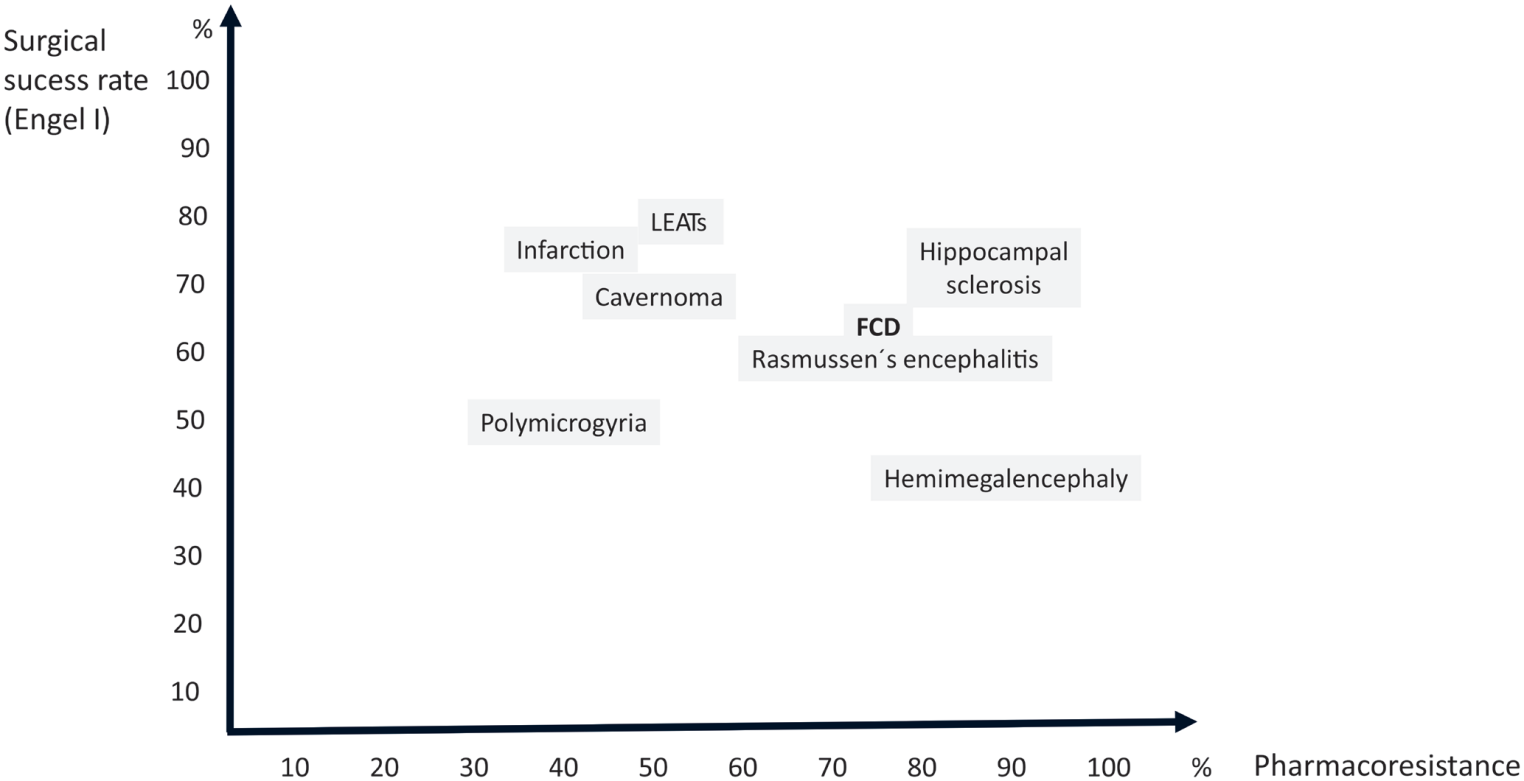
Drug resistance vs. surgical success, including outcome data.^224^, ^225^

The relation of drug resistance vs. surgical success may influence decisions for early or late epilepsy surgery (Figure 19). Aside from the surgical success rate, this also reflects also etiology‐dependent response to pharmacotherapy, with particularly high degrees of drug resistance in patients with hippocampal sclerosis and cortical dysplasia,^25^, ^145^ but may also be assumed also to represent the more general spectrum of etiologies underlying structural epilepsy.

Cognitive outcomes following epilepsy surgery are affected by the underlying etiology as well. Epileptogenic malformations of cortical development may carry motor and cognitive functions with risks of impairment following lesion‐oriented resections.^146^, ^147^ Etiologies with very early manifestation of epilepsy and associated developmental encephalopathy can induce irreversible impairment of cognitive development.^32^, ^148^ In Rasmussen's encephalitis, surgery before the age of 5 years offers the best chances for complete language shift to the primarily non‐dominant hemisphere.^149^ Overall, delayed surgery due to difficult‐to‐localize etiologies has been hypothesized to put patients at a higher risk of cognitive losses following epilepsy surgery.^150^

Appropriate surgical approaches depend not only on the localization and extent of epileptogenic brain lesions but also reflect specific etiology‐dependent aspects of epileptogenicity. Etiology determines the degree of intrinsic epileptogenicity of a lesion and influences the resection size: Outcome studies suggest that FCDs and LEATs need to be resected completely in order to achieve seizure freedom,^151^, ^152^, ^153^ and milder forms of dyslamination surrounding the MR‐visible part of the dysplastic cortex may require invasive recordings or intraoperative ECOG to determine resection borders based on interictal spiking or high‐frequency oscillations.^154^ It is less clear if epileptogenic hippocampi need a complete resection. In individual patients, a second resection of a posterior remnant of a sclerotic hippocampus may lead to complete seizure control. However, prospective studies correlating seizure freedom with the length of the hippocampal resection have failed to prove that complete resections are more favorable.^155^ Particularly in polymicrogyria, appropriately targeted partial resections may suffice to achieve seizure control.^156^, ^157^ The role of areas of FCD type III adjacent to lesions like LEATs for epileptogenesis and for surgical planning so far remains a matter of debate.^158^ Selective microsurgical approaches and minimally invasive selective surgical approaches using laser interstitial thermal therapy^159^ or SEEG‐guided radiofrequency ablation^160^ rely on well‐delineated morphological lesions (e.g., hippocampal sclerosis, hypothalamic hamartomas, or nodular heterotopias) and aim at a maximal preservation of intact brain tissue. Of note, selected multilesional etiologies like cavernomatosis and tuberous sclerosis offer surgical options provided that there is one identifiable epileptogenic cavernoma or leading tuber.

## 5 ESTIMATING RISKS AND BENEFITS OF SURGICAL THERAPY BASED ON MULTIMODAL WORK‐UP

The goal of epilepsy surgery is to improve patients' lives by reducing or eliminating their seizures, while ensuring they can function more comfortably and productively. This requires balancing the risks of complications with the chances of seizure control, using all available data for informed decision‐making. With extensive presurgical testing, objective approaches to integrate multimodal data and improve outcome prediction are a logical step.

### 5.1 Individualizing surgical outcome prediction

Recent advancements, such as nomograms and online risk calculators, have transformed the way outcomes of epilepsy surgery are predicted. Tools like those available at https://www.riskcalc.org offer^115^, ^116^, ^117^, ^118^, ^119^, ^161^, ^162^ individualized forecasts of both seizure control and cognitive outcomes using inputs limited to select clinical characteristics (e.g., age, gender, epilepsy duration) and simplified interpretations of presurgical tests (e.g., MRI findings classified as normal vs. abnormal; scalp EEG seizure localization classified as “always localizable” vs. “sometimes non‐localizable”; and scalp spikes on scalp EEG classified as “absent” vs. “>80% unilateral” vs. “bilateral”). Their predictive accuracy, measured by concordance indices, is moderate to high, ranging between 0.72 and 0.81.^163^ For context, a concordance index of 1 indicates perfect prediction, while 0.5 suggests a result no better than random chance. The seizure freedom score (SFS) simply categorizes the presence of an MRI lesion, seizure frequency (> 20/month), epilepsy duration (>5 years), and presence of convulsions to provide three outcome tiers.^164^ The Epilepsy Surgery Grading Scale (ESGS) takes subcategories of MRI and EEG findings and concordance into account to provide a outcome tiers. The performance of these outcome scores is notably more accurate than predictions made by clinicians based on experience alone, which show a concordance index of only 0.47. Altogether, these tools sacrifice some accuracy for utility and ease of use, facilitating their integration into clinical workflows and providing a scalable scientific approach to aid in more personalized and informed decision‐making.

### 5.2 Progress through advanced computation

Machine learning has also been applied to refine patient selection and enhance outcome predictions. Multiple studies have used these techniques to improve accuracy, indicating potential for even better individualized forecasts.^165^, ^166^, ^167^, ^168^, ^169^, ^170^ However, due to the required sophistication and specialization of data processing and feature extraction, these studies typically apply machine learning to one modality at a time (usually structural MRI, intracranial EEG studies, or functional brain MRI) and so are not benefiting from multimodal data integration and its associated expected prediction benefits.

Recent studies have successfully integrated neuroimaging and neurophysiology data in challenging patients undergoing stereo‐EEG evaluations, some culminating in virtual brain models.^171^, ^172^ These very promising efforts are currently in the research stage, used in select surgical programs with the needed expertise. To reach broader adoption and full clinical integration, an evolution to conquer the following next steps is needed: (1) reproducibility: collaborative research with larger diverse cohorts will address concerns of model overfitting, the risk of falsely high precision in small sample sizes, and the lack of sufficient validation; (2) clinical translatability is now hindered by complex postprocessing to extract key features: innovative computational approaches to automate or simplify the process of feature extraction are needed; and (3) generalizability: models are now generated from data inputs that are not widely available, like research imaging only available in select programs, or intracranial EEG now performed in about half the patients who eventually undergo resection in the US. Expanding the inputs to more available data sources like scalp EEG and structural MRI will expand the impact on the broader epilepsy population. A shift from research to commercialized tools that can be integrated in existing clinical platforms is needed.

## 6 PATIENT MANAGEMENT CONFERENCE

The epilepsy patient management conference is an integral part of an epilepsy center and of the process providing optimal, consensus‐based care for the individual patient considered for a surgical intervention. The updated guidelines for specialized epilepsy centers by the NAEC (National Association of Epilepsy Centers in the United States of America) recommend that all centers should have a formal multidisciplinary, presurgical conference requiring the consistent attendance of a neurosurgeon, neuropsychologist, epileptologists, and neuroradiologist.^36^ A virtual option for the conference can be considered and may not only improve attendance but also facilitate the participation of other specialists when needed or stimulate collaboration with the patient's referring provider.^173^ Each surgical candidate should be presented, including patients considered for neurostimulation such as vagal nerve stimulators. The guidelines emphasize that the patient should be offered the best procedure to improve their seizure control regardless of the center's ability to perform this procedure. If not, a referral to a more suitable center should be offered.

The goal of the conference is to provide a consensus process determining if the patient is a suitable surgical candidate, identifying the best procedure given all available information, and estimating the chance of success and potential risks of the procedure. During the conference, the patient's seizure and past medical history and treatment are presented.^174^ The recent epilepsy monitoring unit evaluation allows the team to review the patient's semiology and EEG findings ideally on the acquisition system to facilitate reformatting and to review the electroclinical correlation of the patient's events. This is typically followed by an unblinded review of structural and functional imaging data, MRI, PET, SPECT, fMRI, magnetic source imaging/electrical source imaging (MSI/ESI), and if available, advanced morphometric or quantitative analysis of the imaging data (Figure 1). The comprehensive assessment includes a review of the neuropsychological and psychosocial findings and any other pertinent information. The group recommendations should be explicitly documented, including the preferred procedure or any alternative options, the need for additional testing, and the consensus estimate of outcome and risk.

Similarly, for patients with incongruent presurgical findings who are candidates for further invasive evaluation, the conference should provide a hypothesis, a preliminary implantation strategy, the therapeutic procedures under consideration, and specify any additional testing required prior to the invasive implantation. The group should also estimate the chance that the invasive procedure provides sufficient data to move forward and at what risk.^129^ Particularly for patients undergoing SEEG evaluation, additional meetings with a smaller group are necessary to propose an implantation scheme to the neurosurgeon and review the final trajectories together with the surgeon.

After the conference, the primary epileptologist checks in with the patient and family to communicate the recommendations and predictions. This is followed by a visit with the surgeon to discuss the details and technical aspects of the procedure, the timeline of surgery, the anticipated length of the procedure, the hospital stay, and recovery. Once informed consent is obtained, the neurosurgical team takes over the presurgical planning and medical clearance. Ideally, the appointment with the epileptologist and surgeon can be offered jointly and complemented by a surgical nurse coordinator who can provide additional support and education, which is particular for complicated invasive procedures.

### 6.1 Case study: Patient counseling and decision making

**FIGURE 20:**
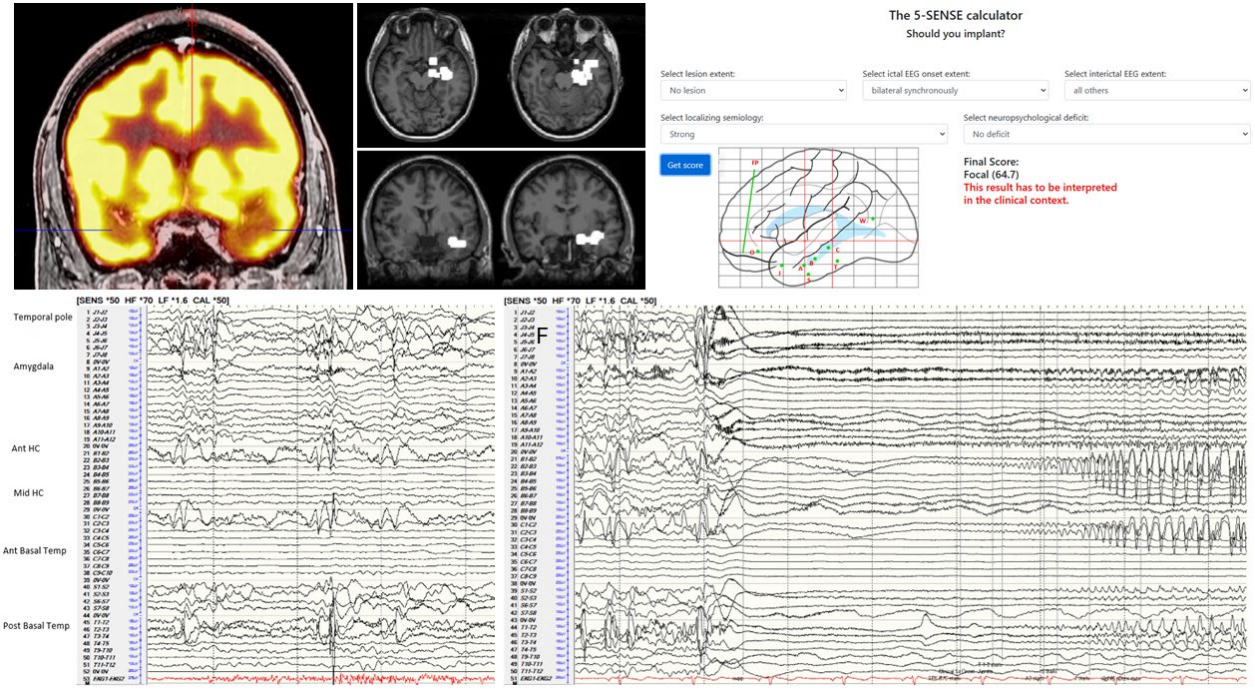
Case study: Counseling and decision‐making.

A 29‐year‐old right‐handed female speech pathologist with a medical history significant for depression and refractory left TLE, onset age 12 years. Failed four ASMs. No specific risk factors for epilepsy. Semiology consisting of focal impaired consciousness seizure with apnea, brief aphasia followed by automatism and unresponsiveness, and FBTC preceded by right head version but a sign of four with left arm extension. MRI was normal, PET with subtle decreased activity within the left anterior temporal lobe (Figure 20A), left dominant for language and memory by fMRI and Wada, without neuropsychological deficits. MEG showed dipolar and cortically constrained distributed sources localizing to the left mesial temporal lobe (Figure 20B). Video‐EEG showed left anterior temporal IED and bilateral temporal seizure onset. SCS was 3, not supporting a direct resection. 5‐Sense score (Figure 20C) predicted a 64.7% chance that an SEEG evaluation would provide useful information, largely driven by the strong localizing semiology.

Patient was counseled that the most likely outcome of the SEEG evaluation would be showing involvement of the hippocampus (~70–80%) with a high risk for memory decline with resection or ablation of the hippocampus. Since a significant memory deficit was not acceptable for her, this could then result in the placement of a neurostimulator rather than a potentially curative procedure.

Patient underwent SEEG implantation (Figure 20D) which showed two spike populations, limited to the hippocampus and synchronized over the left temporal tip and amygdala (Figure 20E) with high frequency oscillations over the latter contacts. EEG onset was seen with DC shift and low voltage fast over the temporal pole and amygdala with a fast propagation over the anterior lateral temporal cortex (Figure 20F). Habitual seizures were elicited from the amygdala and anterior hippocampus with high frequency stimulation.

Patient was offered a selective temporal pole removal and amygdalotomy either through an open resection or LITT. She was quoted a chance of 40–50% seizure freedom after two years with an open resection and 20%–30% with LITT, given that despite the favorable onset pattern, only part of the SOZ would be removed.^175^ She was made aware of the risk of temporary word‐finding difficulties with either procedure. Patient chose LITT as a first step and has been seizure‐free for 2 years.

The case illustrates the process of decision‐making, including a Phase 2 invasive exploration and the value of a group consensus. It also shows the challenges of providing an individualized estimate of success and risk, given the complexity of available data and procedures.

## 7 POSTSURGICAL FOLLOW‐UP

### 7.1 Risk and benefits associated with drug management after epilepsy surgery

Postoperative ASM management starts well before epilepsy surgery. A strong motivation of many persons with epilepsy undergoing surgery is to achieve postoperative seizure freedom in the presence of ASM tapering. At least a reduction of postoperative drug load is asked for.^176^, ^177^, ^178^ However, physicians frequently do not offer surgery with the goal of ASM withdrawal but rather to achieve meaningful seizure control in DRE. In adults, weaning ASMs postoperatively may not change quality of life.^179^ Therefore, realistic expectations regarding postoperative treatment on both sides need to be discussed before the resection.

More than half of the adults with DRE who undergo resective surgery will achieve postoperative seizure freedom at least for some years.^180^ After successful surgery, the patient and the treating physicians need to decide whether and when ASM should be reduced or even withdrawn. Continuation of ASM treatment may have continuing side effects, including potential teratogenicity. Conversely, ASM reduction or withdrawal may increase the risk of seizure recurrence accompanied by the risk of injuries, loss of driving ability, and other drawbacks in private and professional life. Moreover, the rates of seizure freedom tend to decrease with time,^26^, ^181^, ^182^ which is at odds with potential ASM reduction.

Nevertheless, ASM decrease after surgery can be successful and is frequently guided by good surgical outcomes and patient preferences.^177^, ^178^ Detailed recommendations for ASM treatment after epilepsy surgery are lacking, and its management appears to be highly individualized. In clinical practice, persons with epilepsy who remained seizure‐free after the resection for 1–2 years are frequently offered the opportunity to withdraw ASM,^183^ although this may impose an increased risk of seizure recurrence for some patients. In children, ASM taper is attempted in a larger proportion and earlier than in adults, given the limited impact of postoperative seizure freedom duration on recurrence risk and the emphasis on cognitive development. Guidelines indicate that the presence of postoperative IEDs in children increases the risk of seizure recurrence.^179^

Several studies focused on identifying risk factors for seizure recurrence when tapering ASM after successful epilepsy surgery.^26^, ^184^, ^185^, ^186^, ^187^, ^188^, ^189^ Frequently identified risk factors comprise:

- Short time between surgery and start of ASM withdrawal
- Interictal epileptiform discharges in postoperative EEG
- Longer duration of epilepsy
- Incomplete resection
- Number of ASM at time of surgery
- History of focal to bilateral tonic–clonic seizures before surgery
- Seizures before ASM withdrawal

These risk factors may provide assistance for the individual decision to continue or taper ASM. In addition, these data may suggest conceptually that there may be a 3rd outcome category besides cure by or failure of epilepsy surgery. This 3rd category may include patients in whom epilepsy surgery converts their DRE into a drug‐responsive focal epilepsy. These patients may require long‐term postoperative ASM treatment to warrant seizure freedom.

The location and type of procedure may have an additional impact. After seizure recurrence following postoperative ASM withdrawal, most patients undergoing temporal lobe surgery regain seizure freedom when ASM treatment is reintroduced.^190^, ^191^ The chances of seizure freedom appear to be less favorable in this situation after extratemporal surgery.^185^ LITT for DRE may not only have a lower initial success rate, but seizure freedom also seems to fade quicker with a 25% faster drop between year 1 and year 2 outcomes compared with open resection.^192^

### 7.2 Repeat surgery

**FIGURE 21:**
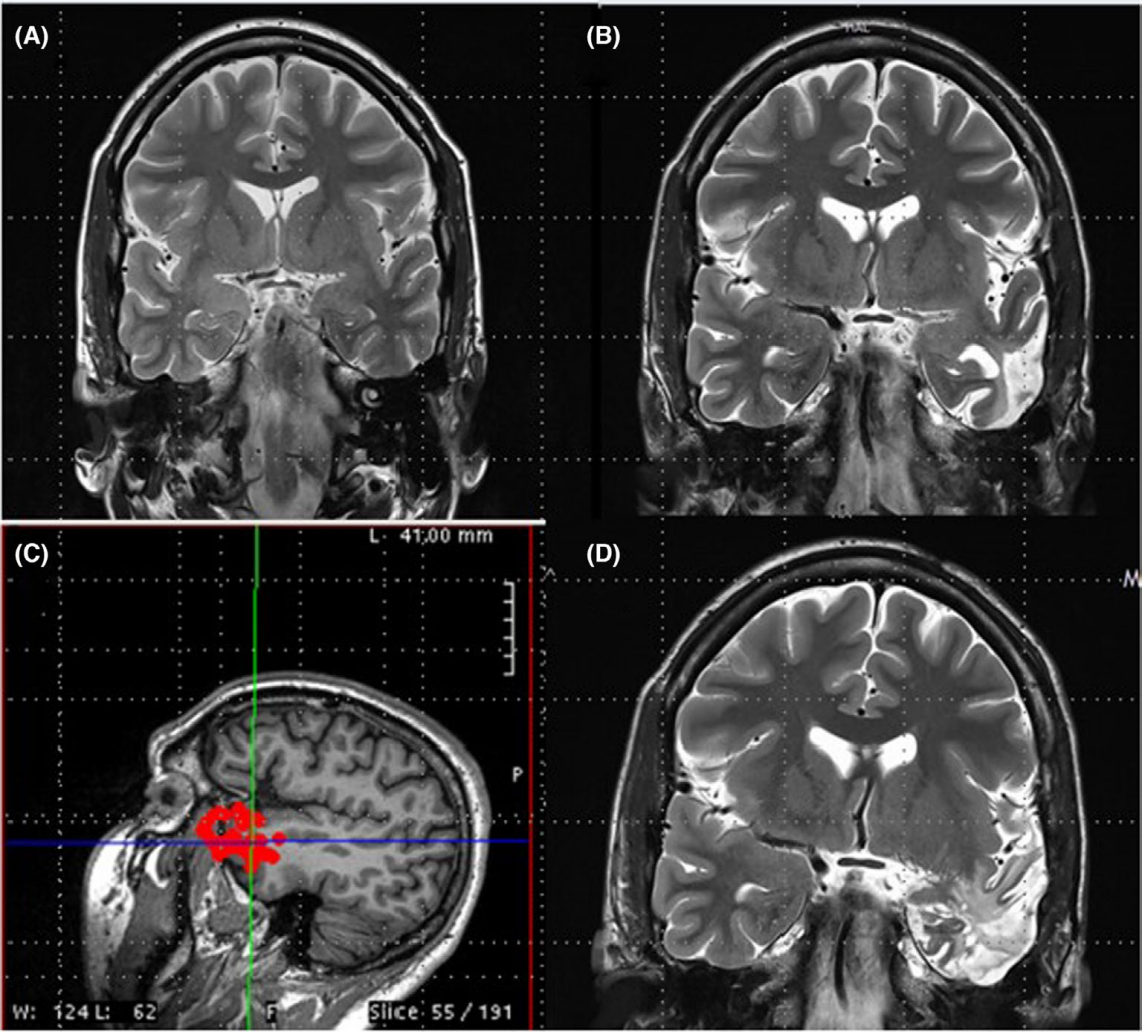
DS, 27 years old at first surgery, right‐handed. (A) The patient suffered from MRI‐negative left temporal lobe epilepsy. The first presurgical work‐up revealed interictal and ictal EEG findings pointing to the left temporal pole. (B) This led to the first surgery consisting of partial resection of the left temporal pole, sparing the mesial structures; histopathology revealed dysplasia IIA. The patient continued to have seizures. (C) MEG source imaging of interictal epileptiform discharges pointed to the left temporal pole, and second non‐invasive presurgical diagnostics revealed congruent EEG findings still suggesting left anterior temporal epilepsy. (D) Second surgery comprised completion of resection of the left temporal pole and resection of the hippocampus head and amygdala. The patient remained seizure‐free after the second surgery.

DRE is a severe disorder that can be challenging to treat. In this situation, epilepsy surgery is known as a valid option for these patients to still achieve seizure freedom.^26^ However, if the first resection fails, treatment becomes even more difficult and frustrating. Therapeutic options then comprise optimizing ASM treatment, stimulation methods, or second surgery. Repeat surgery for intractable epilepsy can be efficacious in selected patients (Figure 21). In order to choose the best treatment for the individual patient, it is important to correctly identify those patients who may have good chances of success with a second surgery.

The presurgical work‐up for a second surgery follows similar lines as the presurgical diagnostics for the initial surgery.^13^, ^193^ This includes renewed interictal and ictal EEG recordings, repeat imaging, and neuropsychological testing. In addition, non‐invasive source imaging, such as magnetoencephalography (MEG), is frequently helpful to localize remaining epileptogenic tissue.^193^ A relevant subset of these patients may also undergo invasive monitoring to design a second resective strategy.

Most studies reported seizure‐free rates after repeated surgery of around 40 to 50%,^194^, ^195^, ^196^ although some studies revealed even higher rates of up to 71%.^193^, ^197^, ^198^, ^199^, ^200^ These findings, however, need to be interpreted with caution because the studies may have been influenced by selection and/or publication bias.

Factors which have been frequently associated with good outcome after repeat surgery were congruent EEG findings, lesional epilepsy/positive MRI, and failure of first surgery due to surgical limitations.^194^, ^195^, ^196^ The need for invasive EEG was found to be a risk factor for failure of repeated epilepsy surgeries.^195^ A study that included patients with several epilepsy surgeries noted a correlation between an increasing number of previous surgeries and worsening of seizure outcomes.^196^

Not surprisingly, many of the associations identified for repeat surgery resemble those that predict good or bad outcomes after the initial epilepsy surgery. The vast majority of the studies were guided by the assumption that a second surgery should address epileptogenic tissue that was left behind and therefore can be successful when the remaining epileptogenic zone is accurately localized and completely resected. However, this concept has been recently challenged by studies suggesting that inherent, potentially genetic characteristics increase the risk of surgical failure regardless of the completeness of the SOZ resection.^201^, ^202^ In a meta‐analysis on repeat epilepsy surgery, the complication rate was around 20%,^195^ but the rates varied widely across studies, likely related to the great spectrum of resective procedures applied and differences in defining postoperative complications.

### 7.3 Neurologic and psychiatric complications of epilepsy surgery

#### 7.3.1 Neurologic complications

Neurosurgical complications can be divided into expected (e.g., right superior quadrantanopia following a left temporal lobectomy) and unexpected (e.g., right homonymous hemianopia associated with a left anterior choroidal artery occlusion).^203^ No neurosurgical procedure is free of potential risks, but if these are “minimal,” the procedure can be considered. Neurologic complications depend on the type of procedure, proximity to eloquent areas, presurgical functional status of specific neurologic and cognitive functions, and include motor and sensory (mono/hemi sensory/hemineglect) deficits, visual field defects, cranial nerve deficits, and cognitive decline (e.g., language, memory).

Except for visual field defects and some cognitive deficits (see below), most neurologic complications are transient and remit within weeks to months following physical, occupational, and speech therapy. Permanent neurologic complications have been reported to range from 2.2% in a population‐based study to 4.7% in a systematic review, with motor deficits ranging between 1% and 1.8% more often following extratemporal resections.^204^, ^205^

Visual field deficits are the most common neurosurgical complications, resulting from anterior temporal lobectomy (aTL), with prevalence rates ranging from 12.9% to 69%.^205^ The vast majority consist of homonymous superior quadrantanopia with no functional implications. However, in 3% of patients, TL with more posterior resections may result in the visual loss in more than one visual quadrant with significant functional implications requiring occupational therapy to learn to scan the visual fields and may also preclude the restoration of driving privileges in patients who become seizure‐free.

TLs have also resulted in cognitive deficits including language and memory disturbances. A systematic review found transient and permanent language deficits in 3.7% and 0.8% of patients, respectively^205^; this review, however, does not address the long‐lasting anomia affecting 25% to 60% of patients following resection of the basal temporal language area in the dominant hemisphere.^203^ The use of laser ablation of mesial temporal structures has decreased the frequency and severity of language deficits.^206^

Verbal memory decline is an expected complication in up to 60% of patients undergoing resection of mesial temporal structures (hippocampus and entorhinal cortex) and parahippocampal gyrus in the dominant hemisphere, particularly in patients whose presurgical neuropsychological testing reveals adequate memory processing.^207^ Verbal memory decline can also be encountered in 20% of non‐dominant mesial temporal resections.^106^ Sparing or limiting the resection of the hippocampus may lower the severity of the deficits. While laser ablation has been reported to be associated with a less frequent and less severe deficit in verbal memory processing, it may occur in patients with presurgical intact verbal memory processing.^208^ Visual memory deterioration can also be expected with resection in the non‐dominant hemisphere and has been reported in 20% to 40%, which may interfere with the recognition of faces and processing complex visual spatial memory processing.

#### 7.3.2 Psychiatric complications

Psychiatric comorbidities have been found in 40% to 60% of patients who undergo epilepsy surgery, the majority of which consist of a mood and/or anxiety disorder, but also include more severe conditions such as psychotic and personality disorders.^209^ Yet, psychiatric aspects of epilepsy surgery remain under‐recognized and poorly investigated and are mostly described for series of TL surgeries. Data vary among studies because of different methodology in the diagnosis of psychiatric disorders, timing of the postsurgical psychiatric evaluation, and postsurgical follow‐up duration. For example, some studies relied on the use of self‐rating screening instruments of psychiatric symptoms (e.g., Beck Depression Inventory‐II), which establish the presence and severity of symptoms of depression over a period of 2 weeks, but do not establish a psychiatric diagnosis of a current and/or past mood disorder and do not identify family psychiatric history, a pivotal variable that is associated with postsurgical iatrogenic risks. Furthermore, several studies limited their investigation to a single psychiatric comorbidity (mostly mood disorder) and failed to identify common concurrent comorbid psychiatric disorders.

The relation between psychiatric comorbidities and epilepsy surgery is complex and can be summarized as follows:

- Presurgical psychiatric comorbidities are associated with an increased risk of postsurgical psychiatric complications, presenting as a recurrence and/or exacerbation of a presurgical condition. Mood and anxiety disorders are the most frequent. For example, a presurgical history of a mood disorder is a strong predictor of postsurgical psychiatric exacerbation, which has been reported in 30% to 40% of patients and develops within the first 3 months after TL, although irritability and mood lability can often be identified from the time of surgery.^210^, ^211^, ^212^ These episodes may improve or remit by 12 months. Likewise, a presurgical history of interictal psychosis is the strongest predictor of a postsurgical psychotic episode.
- A presurgical psychiatric history can have a negative impact on the immediate postsurgical course, leading to an increased need for analgesic medication and longer hospitalizations. Patients with a personality disorder have required hospitalization in a psychiatric unit.^213^ Unfortunately, patients with epilepsy and personality disorders are rarely investigated during presurgical evaluations.
- De novo psychiatric disorders can develop in 10% to 15% of patients following a TL, the majority presenting as a mood and/or anxiety disorder and beginning between 3 and 6 months after surgery.^214^ These may respond to psychotropic medications but may persist after 2 years. In addition, de novo psychotic disorder has been identified in approximately 5% of patients (range: 0%–12%). Most of these psychotic episodes respond to antipsychotic medications and remit completely.^215^
- Remission of presurgical mood and/or anxiety disorders can occur in 30% to 50% of patients with a presurgical history following a TL, particularly in patients who achieve seizure freedom.^216^, ^217^ Likewise, between 25% and 50% of presurgical interictal psychotic disorders were reported to improve.^218^
- Presurgical psychiatric comorbidities may be associated with a lower probability of achieving postsurgical seizure freedom, particularly in patients with complex psychiatric comorbidities, such as personality disorders with and without a mood and/or anxiety and/or psychotic disorders.^219^

Clearly, these data illustrate the need for a comprehensive psychiatric evaluation in every surgical candidate performed by psychiatrists and not derived from neuropsychological evaluations, which complement but do not replace the former. In fact, untreated psychiatric comorbidities may impact the surgical procedure at various levels:

- They may interfere with the patients' ability to collaborate during the presurgical evaluation, understand the benefits and limitations of the surgical procedure, cause patients to refuse the surgical procedure, and/or provide an objective informed consent.
- Knowledge of the presurgical psychiatric history can help anticipate postsurgical risks and prevent them with the introduction and/or adjustment of psychotropic drugs prior to or following the surgical procedure.
- Impact the decision to discontinue ASMs with mood stabilizing and/or anxiolytic properties in patients in whom these drugs may have played a therapeutic effect in the treatment of mood and/or anxiety disorders.
- May impact the perception of severity of cognitive difficulties, including concentration and memory processing.

### 7.4 Rehabilitation

Postsurgical rehabilitation requires a multidisciplinary strategy that is tailored to the individual patient and aimed at addressing neurosurgical, physical, cognitive, psychiatric, and psychosocial complications.^220^, ^221^, ^222^, ^223^ This process, however, should be started during the presurgical evaluation with the aim of understanding the impact of the surgical procedure on the family dynamics, academic and/or employment potential, potential cognitive deficits, and psychiatric complications.

Presurgical rehabilitation should include:

- A vocational evaluation and training for patients who are not working or in school.
- An analysis of the family dynamics to understand a proper adjustment of the different family members to a seizure‐free life of the patient. In fact, postsurgical divorce is not unusual.
- Cognitive rehabilitation of current cognitive deficits.
- Psychiatric management of current psychiatric comorbidities with psychotherapy and/or pharmacotherapy, as behavioral treatments are generally preferable to pharmacotherapy when there is strong evidence of efficacy and non‐inferiority.

Postsurgical rehabilitation should include:

- Tailored application of physical, occupational, and speech therapies to overcome the neurosurgical complications listed above.
- Use of cognitive rehabilitation programs to assist the patient in compensating for any cognitive deficits or declines (e.g., memory, language, executive function). These programs must be tailored to the patient's cognitive/psychiatric deficits and include strategies that rely on the use of mnemonic devices, spaced repetition, and external memory aids such as calendars, planners, and smartphone apps, to name a few. These programs are well established for stroke and traumatic brain injury but have only more recently been applied to epilepsy, with limited data on their efficacy.^220^, ^221^, ^222^ For example, in a review of four studies, Mazur‐Mosiewicz concluded that cognitive rehabilitation yields limited improvement in verbal memory following left temporal lobectomies.^220^ In a review of the literature, del Felice et al. concluded: “Overall, there was insufficient evidence to make definitive conclusions regarding the efficacy of traditional memory rehabilitation strategies, brain training, and non‐invasive brain stimulation.”^222^ Baxendale proposed the concept of “prehabilitation” in surgical candidates whereby patients who are at risk of developing postsurgical cognitive deficits are provided with compensatory strategies before the surgical intervention while their cognitive functions have not been affected “in preparation for postoperative changes.”^223^

## 8 CONCLUSION

This Seminars in Epileptology article aims to provide fundamental principles of the presurgical evaluation of patients with DRE. The goal is to provide a comprehensive, practical learning tool for all trainees in epilepsy and clinical neurophysiology. The main focus is on the advanced techniques applied during a presurgical evaluation, involving neurophysiology, imaging, and neuropsychological assessment, guided by the suspected underlying pathology. In addition, the article discusses the reasoning behind surgical intervention and provides guidance through the process of the epilepsy patient management conference, decision‐making, and subsequent postoperative care and rehabilitation.

## CONFLICT OF INTEREST STATEMENT

BF has received speaker honoraria from UCB, Eisai, and Paladin labs, outside the submitted work. She is an Associate Editor for Epileptic Disorders, the Journal of Clinical Neurophysiology, and the Journal of Sleep Research. ASB has received honoraria for lectures or advice from Angelini Pharma, Desitin, EISAI, JAZZ Pharma, Livanova, Precisis, UCB, and UNEEG. SUS received honoraria for speaker bureau activity and advisory board engagements with Jazz, Neurelis, SK Life Science, and UCB. He is Editor‐in‐Chief of the Journal of Clinical Neurophysiology and Associate Editor of Clinical Neurophysiology and Frontiers Neurology. HMH has served on the scientific advisory board of Angelini, UniQure, Eisai, Jazz, Longboard, and UCB Pharma. He served on the speakers' bureau of or received unrestricted grants from Angelini, Ad‐Tech, Alnylam, Bracco, Desitin, Eisai, Jazz, LivaNova, Nihon Kohden, Pfizer, and UCB Pharma. RMB serves as Associate Editor for Epilepsy & Behavior and Neuropsychology Review. RS has received personal fees as speaker or for serving on advisory boards from Angelini, Arvelle, Bial, Desitin, Eisai, Jazz Pharmaceuticals Germany GmbH, Janssen‐Cilag GmbH, LivaNova, LivAssured B.V., Novartis, Precisis GmbH, Rapport Therapeutics, Tabuk Pharmaceuticals, UCB Pharma, UNEEG, and Zogenix. GR has received payments to her institution from Eisai, Jazz Pharma, Neurax Pharm, Neurocrine, UCB, and Takeda. She is an Associate Editor for Epileptic Disorders, Scientific Reports, and Clinical Epileptology.

**Test yourself'**

1. What is one of the key benefits of performing epilepsy surgery early in life?

- Lower risk of surgical complications
- Higher likelihood of seizure freedom and improved quality of life
- Reduced need for medication postsurgery
- Increased incidence of pharmacoresistance

2. Which of the following statements is wrong?

- Implantation is indicated in all patients where phase 1 presurgical evaluation does not allow to proceed to direct surgery
- Anatomo‐electroclinical correlation integrates the anatomical, electrophysiological, and clinical data to identify the epileptogenic zone
- The 5‐SENSE score is a valuable tool to assist clinicians to identify patients in whom SEEG will allow to identify a focal epileptogenic zone
- Limited sampling is an inherent limitation of invasive EEG
- Bilateral SEEG is negatively correlated with favorable surgical outcome

3. Which of the following statements regarding video‐EEG recording and interpretation is correct:

- It is sufficient to record one seizure for presurgical evaluation
- Antiseizure medication is usually discontinued on the day of admission to the epilepsy monitoring unit
- Seizures in scalp EEG can be falsely lateralizing
- Early ictal tachycardia is suggestive of temporal neocortical seizures
- Central apnea is a localizing sign for precuneus seizures

4. Which of the following factors is a positive prognostic factor for good surgical outcome?

- Focal to bilateral tonic–clonic seizures
- Bilateral independent interictal epileptiform discharges
- MRI with focal cortical dysplasia type II

- III is true, I + II are false
- I + II are true, III is false
- I is true, II and III are false.
- II is true; I and III are false
- All are true

5. Which aspects of epilepsy surgery are influenced by the underlying etiology?

- Surgical outcome
- Surgical approach
- Surgical risks
- Degree of pharmacoresistance
- All are correct

6. According to this article, the following represent key challenges hindering the clinical integration of machine learning algorithms predicting surgical outcomes:

- Lack of large‐scale external model validation
- Complex requirements for data processing
- High demands for computational infrastructure are needed for implementation
- Insufficient physician trust
- All of the above

7. According to this article, a multidisciplinary Epilepsy Management conference is recommended for the following surgical procedures:

- Resective or ablative surgery
- Responsive neurostimulation
- Deep brain stimulation
- Vagal nerve stimulator implant
- All of the above

8. Which of the following is NOT a risk factor for seizure recurrence with postoperative ASM tapering?

- Short time since surgery or seizures before start of ASM withdrawal
- Interictal epileptiform discharges on postoperative EEG
- Short duration of epilepsy
- Incomplete resection
- History of focal to bilateral tonic–clonic seizures before surgery

9. Which of the following statements regarding postoperative complications is correct?

- Permanent neurologic complications have been reported to range from 1% to 2%
- Prevalence of visual field deficits after TL ranges from 5% to 10%
- In 10% of TL patients, a more extensive visual field deficit beyond the superior quadrant can be seen
- De novo psychiatric disorders can develop in 10% to 15% of patients following a TL
- Verbal memory deterioration is seen in up to 80% of dominant TL surgeries

10. According to this article, which of the following MR sequences is not necessarily part of an epilepsy protocol:

- 3D T1
- 3D T2
- 3D FLAIR
- Coronal T2
- Coronal FLAIR

11. What is the role of a neuropsychological assessment in the context of a presurgical work‐up for pharmacoresistant epilepsy?

- To establish a cognitive baseline prior to surgery
- To estimate risk for postoperative cognitive decline
- To examine the pattern of cognitive performance and consistency with results of other presurgical investigations (e.g., EEG, MRI)
- A and B
- All of the above

*Answers may be found in the* *supporting information*

## Supporting information


Appendix S1.

## Data Availability

The data that support the findings of this study are available from the corresponding author upon reasonable request.
