# Supplementary material for: Seminars in epileptology: Presurgical epilepsy evaluation
Source: Epileptic Disord. 2025 Sep 24;27(6):1105–47. doi: 10.1002/epd2.70105 (PMC12747706; doi:10.1002/epd2.70105)
Supplement: Supplementary file 1 — Appendix S1. [file EPD2-27-1105-s001.docx]

Answers

1. B. Higher likelihood of seizure freedom and improved quality of life.

2. A. Implantation is indicated in patients where phase 1 presurgical evaluation does not allow to proceed to direct surgery, but where non-invasive data still allow to generate a strong hypothesis of a single spatially constrained and potentially operable region.

3. C. Seizures in scalp EEG can be falsely lateralizing. Examples are epileptic generators close to the midline. Early ictal tachycardia is suggestive for anterior insula epilepsy. Central apnea is a localizing sign for amygdala seizures; it is typically observed before first temporal EEG changes are seen in scalp EEG. The number of seizures that need to be recorded before the decision for epilepsy surgery is dependent on the a priori hypothesis. Antiseizure medication should be gradually tapered but not abruptly discontinued at the time of EMU admission to avoid non-habitual seizures and complications from bilateral tonic–clonic seizures.

4. A. Focal cortical dysplasia type II is often associated with a good surgical outcome, whereas focal to bilateral tonic–clonic seizures and bilateral independent interictal epileptiform discharges (different from bilateral temporal interictal epileptiform discharges) have been shown to be more frequently associated with a poor surgical outcome.

5. E. Etiology impacts all four aspects.

6. E. All four aspects are challenges for the integration of Machine Learning.

7. E. A multidisciplinary Epilepsy Management conference is recommended for all listed surgical procedures.

8. C. Long and not short duration of epilepsy is a risk factor for seizure recurrence.

9. D. De novo psychiatric disorders can develop in 10% to 15% of patients following a TL.

Below are the correct percentages for the other complications:

- Permanent neurologic complications have been reported to range from 2 to 5%.
- Prevalence of visual field deficits after TL ranges from 10 to 70%.
- In 3% of TL patients, a more extensive visual field deficit beyond the superior quadrant can be seen.
- Verbal memory deterioration is seen in up to 60% of dominant TL.

10. B. 3D T1 and 3D FLAIR oriented along the ac-pc line and coronal T2 and FLAIR oriented perpendicular to the long hippocampal axis are recommended for an epilepsy MRI protocol but not 3D T2 sequences.

11. E. The role of a neuropsychological assessment in the context of a presurgical work-up for pharmacoresistant epilepsy includes all three aspects: to establish a cognitive baseline, to estimate risk for postoperative cognitive decline, and to examine the pattern of cognitive performance and consistency with results of other presurgical investigations (e.g., EEG, MRI).
